# Hidden nursing complexity within diagnosis-related groups (DRGs): a one-year retrospective study of standardized nursing diagnoses and actions among adult hospitalizations in Italy

**DOI:** 10.1186/s12912-026-04806-6

**Published:** 2026-06-25

**Authors:** Antonello Cocchieri, Fabio D’Agostino, John Michael Welton, Mario Cesare Nurchis, Nursing and Public Health Group, Gianfranco Damiani, Manuele Cesare

**Affiliations:** 1 A. Gemelli IRCCS University Hospital Foundation, Largo Agostino Gemelli 8, 00168 Rome, Italy; 2 Section of Hygiene, Department of Life Sciences and Public Health, Catholic University of the Sacred Heart, Largo Francesco Vito 1, 00168 Rome, Italy; 3 Department of Medicine, Saint Camillus International University of Health Sciences, Via di Sant’Alessandro 8, 00131 Rome, Italy; 4 Division of Health Systems, Leadership, and Informatics, University of Colorado College of Nursing, 13120 East 19th Avenue, Aurora, CO 80045 USA; 5 Department of Life Science, Health and Health Professions, Link Campus University, Via del Casale di S. Pio V 44, 00165 Rome, Italy; 6 Hospital Hygiene Unit, A. Gemelli IRCCS University Hospital Foundation, Largo Agostino Gemelli 8, 00168 Rome, Italy

**Keywords:** Diagnosis-related groups, Outliers, DRG, Standardized nursing terminology, Length of stay, Prospective payment system, Reimbursement mechanisms

## Abstract

**Background:**

Diagnosis-related groups (DRGs) are used within prospective payment systems to classify hospitalizations and standardize reimbursement but may insufficiently capture nursing complexity. This study aimed to describe the variability in nursing complexity within the most prevalent DRGs among adult inpatients and to examine its relationship with DRG-specific length of stay thresholds and DRG weight.

**Methods:**

Adult hospitalizations discharged in 2022 from a large acute-care hospital in Rome, Italy, were analyzed. Nursing complexity was measured using counts of nursing diagnoses documented within 24 hours of admission and nursing actions recorded throughout hospitalization. Variability within and across DRGs was explored descriptively. Comparisons were conducted between hospitalizations within and exceeding DRG–specific length of stay thresholds. Linear regression models examined the proportion of variability in nursing complexity explained by DRG weight, both before and after adjustment for demographic and clinical variables.

**Results:**

The study included 14,169 hospitalizations across the 20 most frequent DRGs. Marked variability in nursing diagnoses and nursing actions was observed not only between DRGs but also within the same DRG, with counts ranging from 1 to 17 for nursing diagnoses and from 1 to 770 for nursing actions. Patients classified as medically similar under the same DRG exhibited substantial heterogeneity in nursing complexity. Hospitalizations exceeding DRG–specific length of stay thresholds showed consistently higher nursing complexity compared to those remaining within expected limits, both in terms of nursing diagnoses (mean 7.5, SD 4.3 vs. 4.0, SD 2.5; Welch’s t(265.2) = −13.0, *p* < 0.001; Cohen’s d = 1.0, 95% CI 0.9–1.1) and nursing actions (median 50, IQR 45 vs. 17, IQR 10; Mann-Whitney *U* = 311249, Z = −23.1, *p* < 0.001; *r* = 0.19). In univariable models, DRG weight showed modest associations with nursing diagnoses (β = 0.140, *p* < 0.001; R^2^ = 0.020) and nursing actions (β = 0.100, *p* < 0.001; R^2^ = 0.010). In multivariable models, the magnitude of these associations was markedly reduced, and DRG weight remained weakly associated with both nursing diagnoses (β = 0.030, *p* = 0.001; model R^2^ = 0.130) and nursing actions (β = −0.018, *p* = 0.040; model R^2^ = 0.097).

**Conclusions:**

DRGs do not adequately capture nursing complexity. Integrating standardized nursing data into hospital resource utilization and risk-adjustment models may improve the visibility of nursing care and support more accurate resource allocation in hospital financing systems.

**Supplementary information:**

The online version contains supplementary material available at 10.1186/s12912-026-04806-6.

## Purpose

The present study aimed to examine variability in nursing complexity within the most prevalent DRGs among adult inpatients in an acute-care hospital.

The specific objectives were to: (*A*) describe the distribution and characteristics of the most frequently assigned DRGs; (*B*) describe patient and hospitalization characteristics by DRG; (*C*) analyze differences in nursing complexity, measured through nursing diagnoses and nursing actions, among patients classified within the same DRGs; (*D*) compare nursing complexity between hospitalizations exceeding DRG-specific length of stay (LOS) thresholds and those remaining within expected limits; and (*E*) quantify the association between DRG weight and nursing complexity indicators.

**Supplementary information:**

The online version contains supplementary material available at 10.1186/s12912-026-04806-6.

## Introduction

Diagnosis-related groups (DRG) systems are designed to capture a diagnosis, a procedure, and an expected cost [1]. However, they do not capture how illness is experienced in the hospital setting, nor how nurses continuously manage its evolving risks and care demands at the bedside. Within a single DRG code and fixed reimbursement, the administrative classification assumes clinical and economic homogeneity, yet it remains silent on the day-to-day intensity through which care is enacted.

When this variability is compressed into a single fixed payment unit, nursing complexity becomes structurally invisible within hospital finance [2], perpetuating a structural fracture that has characterized nursing reimbursement for over a century [3].

## Background

### Summary of existing literature and research gap

DRGs were originally developed in the United States during the 1970s as a case-mix classification system to describe hospital output and compare resource use across clinically similar patient groups; they were subsequently incorporated into prospective payment systems, most notably Medicare in the early 1980s [4].

By design, DRGs classify hospitalizations using a combination of clinical and demographic variables—such as principal and secondary medical diagnoses, medical procedures performed, patient age and sex, and the presence of comorbidities and complications—with the aim of grouping patients with relatively homogeneous expected resource consumption [5]. Within DRG-based reimbursement systems, this expected resource consumption is generally modeled through predefined tariffs, relative weights, and DRG-specific length of stay (LOS) thresholds; exceeding these thresholds is commonly interpreted as an operational signal of atypical complexity and potentially greater hospital resource use [6]. Today, DRGs are the foundation of hospital prospective payment systems in many high-income countries, serving to classify admissions, standardize reimbursement, and benchmark performance [1, 7].

Although the general logic of DRG-based classification is shared internationally, its implementation varies across healthcare systems according to national reimbursement rules, regulatory frameworks, costing methods, and organizational characteristics [8–12]. In Italy, since January 1st 1995, DRGs have been adopted within the National Health Service as the standard framework for the classification and reimbursement of acute hospital admissions, with nationally defined tariffs, relative weights, and LOS thresholds; the system has evolved over time through successive DRG versions, including version 10.0 (1994–2005), version 19.0 (from 2006), and version 24.0 (from 2009) [13].

Within this framework, patients grouped within the same DRG are assumed to have broadly comparable levels of clinical severity and resource needs [14]. In practice, however, DRG-reimbursement reflects a fixed economic value per case that is largely independent of day-to-day variation in patient care needs during hospitalization [15, 16]. As a result, these systems operationalize a definition of case complexity primarily grounded in biomedical criteria, leaving nursing care demands largely unmeasured and economically invisible [2, 3, 17].

At the bedside, nurses routinely observe that patients assigned to the same DRG differ substantially in clinical risk, dependency, and care needs, including surveillance, symptom management, education, and discharge coordination [18]. Such heterogeneity is often driven by pre-existing conditions—such as chronic disease trajectories and multimorbidity—or different patient responses to acute conditions—such as anxiety and self-care deficits, factors that generate evolving and unpredictable care needs that are not explicitly incorporated into DRG classification logic [15, 18, 19]. Consequently, patients who are medically similar according to DRGs may require markedly different levels of nursing care in terms of intensity and dependency [18, 20].

Nursing complexity, as a multidimensional construct reflecting patients’ care needs and the corresponding nursing workload [15, 21], is inherently sensitive to this variability when explicitly measured, as it emerges from an early and ongoing biopsychosocial assessment, therapeutic interventions, and adaptive responses to patients’ changing needs—processes that more accurately reflect workload, time, and professional expertise at the point of care [22]. Nevertheless, within DRG-based reimbursement frameworks, nursing contributions are typically aggregated into undifferentiated “room and board” charges rather than explicitly measured, limiting their visibility in financial planning and performance evaluation [2, 23]. In this sense, rather than resulting from a lack of nursing data, this limited visibility derives from a reimbursement structure predominantly organized around administratively and medical variables, in which nursing-specific indicators are not explicitly incorporated into case-mix classification and payment determination [3, 15]. This lack of integration is particularly relevant because it implies that a substantial component of care variability remains not only unmeasured, but also unaccounted for in predictive outcome models. Empirical evidence, conversely, suggests that nursing data contribute independently to outcome prediction and, when integrated with DRG-based information, may enhance the explanatory performance of predictive models [16, 24–26].

Concerns regarding the omission of nursing complexity from case-mix classification date back to the origins of the DRG system, whose architects cautioned that this could distort payment accuracy and disadvantage hospitals caring for patients with greater care dependency [27, 28]. These concerns have been reiterated in subsequent nursing and health services research, which has questioned whether medically defined case-mix groups adequately capture variability in nursing complexity [15]. Today, these issues remain relevant in adult acute care, where population ageing and the growing burden of chronic and multimorbid conditions intensify demands placed on nursing practice and daily care activities, including psychosocial support, care coordination, and transitional planning [29].

The increasing availability of electronic nursing documentation and standardized nursing terminologies offers an opportunity to address this structural limitation [30]. Standardized nursing terminologies—such as nursing diagnoses (NDs) and nursing actions (NAs), which reflect nurses’ clinical judgments about patients’ responses to health conditions and the corresponding care activities delivered—enable the measurement of nursing complexity at the patient level, thereby extending case-mix analysis beyond traditional biomedical criteria [15, 26].

Despite these advances, empirical evidence examining within-DRG variability in nursing complexity using routinely collected standardized nursing data remains limited in adult acute-care settings. In addition, the extent to which DRG weight explains variability in standardized nursing complexity indicators remains insufficiently explored. The present study addresses these gaps by examining variability in nursing complexity within the most prevalent DRGs among adult inpatients, comparing nursing complexity according to DRG-specific LOS threshold status, and exploring the association between DRG weight and nursing complexity indicators.

## Methods

### Design, study setting, and sampling

This retrospective observational study used routinely collected administrative, clinical, and standardized nursing data from an adult acute-care hospital in Rome, Italy, with 1,552 curative beds. The study site was selected because it allows linkage between DRG-based administrative data and routinely collected standardized electronic nursing documentation, which was essential for addressing the study objectives.

The study was conducted within the Italian National Health Service, where DRG-based reimbursement is applied nationally as part of a prospective hospital payment system. In this context, DRGs are used primarily for hospital financing and administrative classification, whereas nursing care is not reimbursed as a separate billable component but is embedded within the overall hospitalization tariff.

The hospital is a large tertiary-care teaching institution providing a wide range of medical and surgical services. Nursing care is delivered according to a standardized organizational model, namely Primary Nursing, which has been associated with increased accuracy of nursing documentation [31]. In addition, the hospital operates within an accreditation framework aligned with Joint Commission International (JCI) standards, which promote high-quality clinical documentation in accordance with safety and quality-of-care standards [32].

The study included hospitalizations discharged consecutively between 1 January and 31 December 2022; the unit of analysis was the hospitalization (discharge episode). DRGs were assigned at discharge as part of routine reimbursement and performance reporting. The study was designed and reported in accordance with the REporting of studies Conducted using Observational Routinely-collected health Data (RECORD) statement [33].

### Inclusion and exclusion criteria

The analytic dataset comprised adult inpatient hospitalizations (age ≥ 18 years at admission) with a LOS ≥ 2 days, covering all adult inpatient units across the hospital, including medical, surgical, and intensive care settings. Hospitalizations shorter than 2 days were excluded, as in the study hospital these are typically classified as day hospital or day surgery admissions and are managed through distinct organizational pathways and documentation practices [22]; therefore, they were not considered inpatient episodes for the purpose of this analysis.

A DRG-level eligibility filter was applied to align the sample with the prespecified analytic objectives. Specifically, hospitalizations assigned to DRGs for which no official ministerial LOS threshold is defined in the Italian regulatory framework were excluded, as threshold-based stratification was not applicable for these DRGs [13]. Eligible DRGs were subsequently ranked according to their prevalence within the remaining cohort, and analyses were restricted to hospitalizations classified within the 20 most frequently assigned eligible DRGs to focus on high-volume case-mix categories and ensure stable within-DRG estimates.

### Variables and data sources

Data were derived from multiple institutional sources used for routine clinical documentation and administrative activities. All variables were linked at the hospitalization level. Each variable is reported below together with its specific data source.

### Independent variables

The main independent variable was DRG weight, extracted from the hospital discharge register (HDR) [34]. The DRG weight reflects the expected consumption of healthcare resources associated with a given DRG and is used as an indicator of resource absorption at the hospitalization level, as well as its relative costliness compared with other DRGs within the reimbursement system [35]. In addition, DRG-specific LOS threshold status was used as a grouping variable in comparative analyses. This variable was derived by combining LOS data extracted from the HDR with DRG-specific LOS thresholds obtained from the Italian national regulatory framework [13].

### Dependent variables

The dependent variables were the two indicators of nursing complexity: the number of NDs and NAs. These data were extracted from the Professional Assessment Instrument (PAI), a clinical nursing information system for documenting nursing care processes [25]. The PAI adopts the Clinical Care Classification System (CCC) as the standardized nursing terminology for NDs and NAs [36]. In the PAI workflow, CCC-coded NAs are automatically cross-mapped to the Italian Nomenclature of Nursing Care Performance (INNCP), a locally adopted standardized nursing language, which provides more granular specification of bedside NAs [37]. Accordingly, all NAs reported in this study correspond to INNCP-coded activities.

The PAI includes a validated rule-based clinical decision support algorithm, developed through expert consensus and content validation, which generates evidence-informed suggestions for NDs and NAs based on structured patient assessment data [38]. These suggestions are not prescriptive and require nurses’ active confirmation, modification, or rejection in real time before being incorporated into the clinical record. Therefore, the documented NDs and NAs analyzed in this study reflect nurse-validated standardized documentation, supported by decision support within the clinical workflow.

In this study, we used NDs documented at admission (within the first 24 hours) and all the NAs documented throughout the entire hospital stay. Accordingly, NDs and NAs were conceptualized as complementary but distinct indicators of nursing complexity. NDs documented within the first 24 hours were used to capture the breadth of nurse-identified patient care needs at admission, reflecting baseline complexity. NAs documented throughout hospitalization were used to capture the volume of nursing care delivered over time, reflecting nursing resource absorption. This operationalization is consistent with prior studies that have used standardized nursing data to quantify nursing complexity, characterize patterns across clinical populations, and examine its association with clinically relevant outcomes, including LOS and intensive care unit transfer [15, 21, 39, 40].

### Other variables

Other variables used for descriptive purposes and sample characterization included age, gender, LOS, DRG assignment (version 24.0), and DRG type (medical vs. surgical), all extracted from the HDR. The number of medical diagnoses and procedures was also obtained from the HDR, whereas DRG-specific LOS thresholds and corresponding reimbursement values (costs) were obtained from the Italian national DRG regulatory framework [13, 41]. Clinical variables included the medical diagnoses count and the number of medical procedures recorded for each hospitalization. Medical diagnoses count was defined as the total number of International Classification of Diseases, Ninth Revision, Clinical Modification (ICD-9-CM) diagnosis codes (principal and additional), while procedure count was defined as the total number of ICD-9-CM procedure codes recorded in the HDR for each hospitalization. These variables were used as descriptive indicators of overall diagnostic and procedural burden and were not analyzed at the individual code level.

### Statistical analysis

The analysis combined descriptive components with prespecified comparative analyses to characterize nursing complexity within DRGs and to test a priori differences between hospitalizations within versus exceeding the DRG-specific LOS threshold. Patient characteristics and study variables were first summarized using descriptive statistics appropriate to the measurement level and data distribution, including frequencies and percentages for categorical variables, and means with standard deviations, medians with interquartile ranges, and ranges for continuous or count variables, as appropriate.

Within each DRG, nursing complexity was examined at the individual hospitalization level to capture heterogeneity in NDs and NAs among patients classified as medically similar. Ranges and violin plots were used as descriptive and graphical tools to support the inspection of within-DRG dispersion and distributional features of nursing variables (i.e., NDs and NAs). Descriptive analyses were exploratory, whereas comparisons between hospitalizations within versus exceeding the DRG-specific LOS threshold for the primary nursing complexity indicators (NDs and NAs) were defined a priori based on the study objectives; all remaining analyses should be interpreted as exploratory.

Hospitalizations were stratified based on whether their LOS exceeded the DRG-specific expected threshold. Comparisons focused on differences in nursing complexity indicators (i.e., counts of NDs and NAs). These comparisons were intended to describe the distribution of nursing complexity according to LOS-threshold status and were not designed to assess causal directionality between nursing complexity and LOS. For variables meeting distributional assumptions, mean-based comparisons were conducted (e.g., NDs); when assumptions were not satisfied, rank-based methods were applied (e.g., for NAs, which showed marked right-skewness with a small subset of high nursing-intensity outliers, i.e., extreme but clinically plausible values). Normality was assessed using skewness and kurtosis statistics, with values within ±2 for skewness and ±7 for kurtosis considered indicative of approximate normal distribution [42]. Homogeneity of variances was assessed before mean-based comparisons; when this assumption was not met, Welch’s t test was applied. When distributional assumptions were violated, non-parametric alternatives were used; specifically, the Mann–Whitney U test was applied for comparisons of NAs, given their marked right-skewness and the presence of extreme values. For rank-based comparisons, effect size was quantified as $r = {Z \over {\sqrt N }}$, where *N* is the total number of observations across groups. For mean-based comparisons, standardized mean differences (Cohen’s *d*, non-pooled) with 95% confidence intervals were reported. This approach was used to account for unequal variances and unbalanced sample sizes.    

To assess the proportion of variability in nursing complexity explained by DRG weight, two separate univariable linear regression models were fitted using DRG weight as the independent variable, with ND and NA counts entered as dependent variables in separate models. In addition, multivariable linear regression models were separately estimated by including age, sex, number of medical diagnoses, and number of medical procedures as covariates. Standardized regression coefficients (β) and measures of explained variance (R^2^ and adjusted R^2^) were reported.

The primary comparisons were defined a priori and aligned with the study objectives; therefore, no adjustment for multiple comparisons was applied, consistent with established epidemiological recommendations discouraging routine multiplicity adjustments for pre-specified primary hypotheses [43]. Statistical significance was assessed using two-sided tests with a threshold of *p* < 0.05. Data management, statistical analyses, and graphical outputs were performed using IBM SPSS Statistics® for macOS (version 31.0.0.0) (IBM Corp., Armonk, NY, USA) and R for macOS (version 4.5.1) with the *ggplot2* package (version 3.5.2) [44, 45].

### Data extraction

Following consent procedures, data were extracted at the hospitalization level by the hospital’s information and communication technology unit, de-identified through removal of direct identifiers, and then made available to the research team in de-identified form. The study did not affect patient care.

## Results

### Distribution and structural characteristics of the selected DRGs in the study year (Objective *A*)

**Fig. 1:**
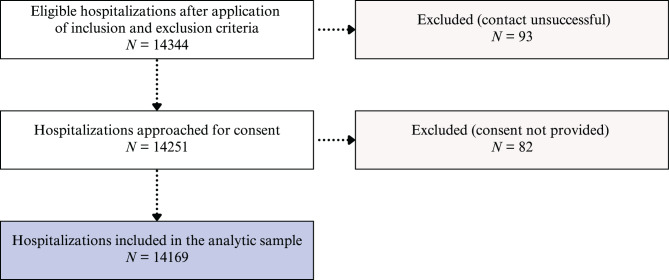
Flowchart of the selection process for the analytic sample. Note: The flowchart illustrates the selection process of hospitalizations included in the study. The unit of analysis was the hospitalization. Exclusions were due to unsuccessful contact or lack of study-specific informed consent

The analytic sample was composed of 14,169 adult hospitalizations which met all inclusion criteria, with no missing data across study variables. A flowchart describing the selection process of the study population, including the number of eligible hospitalizations, patients contacted, and those included in the final analytic sample, is provided in Fig. 1.

The analytic sample covered a broad institutional case-mix (71 admitting units), predominantly from surgical units (*n* = 11,347; 80.1%), followed by medical units (*n* = 2,615; 18.5%) and intensive/sub-intensive care units (*n* = 207; 1.5%). A detailed distribution of admitting units is provided in the Supplementary Materials (Supplementary File 1).

Overall, the selected DRGs showed substantial variability in weight, LOS thresholds, and associated reimbursement values. DRG weights ranged from 0.7204 (DRG 311, *Transurethral Procedures W/O CC*) to 3.1739 (DRG 2, *Craniotomy Age > 17 W/O CC*). DRG-specific LOS thresholds showed marked variability, ranging from 10 days (e.g., DRG 311 and DRG 260, *Subtotal Mastectomy for Malignancy W/O CC*) to 45 days (DRG 570, *Major Small & Large Bowel Procedures W CC Without Major Gastrointestinal Diagnosis*). Corresponding national reimbursement costs for ordinary hospitalizations within DRG-specific thresholds ranged from € 1258 (DRG 500, *Back & Neck Procedures Except Spinal Fusion W/O CC*) to € 11,872 (DRG 2) per episode, as reported in Table 1.

**Table 1:** Distribution and structural characteristics of the analyzed DRGs in the study year (*N* = 14169)

DRG characteristics							Descriptive Statistics	
Code	Description	Category	MDC	Weight	LOS threshold	Cost per episode (€)	N	%
359	Uterine & Adnexa Procedures for Non-Malignancy W/O CC	S	13	0.9785	11	3027	2255	15.9
290	Thyroid Procedures	S	10	0.9978	11	3340	1734	12.2
149	Major Small & Large Bowel Procedures W/O CC	S	6	1.9057	28	7113	892	6.3
288	O.R. Procedures for Obesity	S	10	1.8598	17	5681	805	5.7
494	Laparoscopic Cholecystectomy W/O C.D.E. W/O CC	S	7	0.8890	10	5293	754	5.3
79	Respiratory Infections & Inflammations Age > 17 W CC	M	4	1.7612	40	5744	718	5.1
2	Craniotomy Age > 17 W/O CC	S	1	3.1739	34	11,872	692	4.9
576	Septicemia Age > 17	M	18	1.6432	37	5493	669	4.7
353	Pelvic Evisceration, Radical Hysterectomy & Radical Vulvectomy	S	13	1.7728	22	6203	584	4.1
203	Malignancy of Hepatobiliary System or Pancreas	M	7	1.2825	35	4085	517	3.6
260	Subtotal Mastectomy for Malignancy W/O CC	S	9	0.8896	10	2354	515	3.6
570	Major Small & Large Bowel Procedures W CC Without Major Gastrointestinal Diagnosis	S	6	2.7406	45	11,318	500	3.5
14	Intracranial Hemorrhage or Cerebral Infarction	M	1	1.2605	30	3891	481	3.4
311	Transurethral Procedures W/O CC	S	11	0.7204	10	2350	473	3.3
75	Major Chest Procedures	S	4	2.3960	28	8737	472	3.3
171	Other Digestive System O.R. Procedures W/O CC	S	6	1.6750	31	4498	458	3.2
500	Back & Neck Procedures Except Spinal Fusion W/O CC	S	8	1.2589	14	1258	440	3.1
544	Major Joint Replacement or Reattachment of Lower Extremity	S	8	2.0283	22	8837	424	3
357	Uterine & Adnexa Procedures For Ovarian Or Adnexal Malignancy	S	13	1.7499	27	6791	400	2.8
87	Pulmonary Edema & Respiratory Failure	M	4	1.2243	27	3802	386	2.7

Note: LOS threshold values reported in the table represent the official threshold assigned to each DRG by the Italian national DRG reimbursement framework. MDC are indicated by numerical codes corresponding to major body systems or clinical domains as defined in the DRG classification and represented in the study sample (MDC 1 = Diseases and disorders of the nervous system; MDC 4 = Diseases and disorders of the respiratory system; MDC 6 = Diseases and disorders of the digestive system; MDC 7 = Diseases and disorders of the hepatobiliary system and pancreas; MDC 8 = Diseases and disorders of the musculoskeletal system and connective tissue; MDC 9 = Diseases and disorders of the skin, subcutaneous tissue and breast; MDC 10 = Endocrine, nutritional and metabolic diseases and disorders; MDC 11 = Diseases and disorders of the kidney and urinary tract; MDC 13 = Diseases and disorders of the female reproductive system; MDC 18 = Infectious and parasitic diseases, systemic or unspecified sites). Tariff per episode (€) refers to the national cost for ordinary hospitalizations with LOS > 1 day and within the DRG-specific LOS threshold

Abbreviations: DRG, diagnosis-related group; MDC, major diagnostic category; LOS, length of stay; W/O, without; CC, complications; S, surgical; M, medical; O.R., operating room; C.D.E., Common Duct Exploration; W, with

### Patient and hospitalization characteristics by DRG (Objective *B*)

Patient and hospitalization characteristics varied markedly across DRGs, reflecting heterogeneous clinical profiles and care pathways (Table 2).

**Table 2:** Patient and hospitalization characteristics by DRG

DRG data		Descriptive Statistics					
DRG code	DRG description	Female (n; %)	Age (mean, SD)	Medical diagnoses count (mean, SD)	Medical procedures count (mean, SD)	LOS (median, IQR)	Patients Exceeding LOS Threshold (n; %)
359	Uterine & Adnexa Procedures for Non-Malignancy W/O CC	2255 (100.0)	49.7 (13.4)	1.9 (1.1)	3.3 (1.4)	3 (1)	4 (0.2)
290	Thyroid Procedures	1304 (75.2)	53.2 (14.1)	1.1 (0.4)	2.2 (0.9)	2 (0)	2 (0.1)
149	Major Small & Large Bowel Procedures W/O CC	511 (57.3)	62.0 (14.6)	1.4 (0.8)	4.0 (1.5)	5 (3)	0 (0)
288	O.R. Procedures for Obesity	568 (70.6)	46.6 (11.1)	1.3 (0.9)	3.6 (0.9)	2 (0)	2 (0.2)
494	Laparoscopic Cholecystectomy W/O C.D.E. W/O CC	445 (59.0)	56.9 (14.0)	1.1 (0.4)	2.0 (0.8)	2 (1)	5 (0.7)
79	Respiratory Infections & Inflammations Age > 17 W CC	312 (43.5)	74.2 (15.0)	4.8 (1.5)	4.3 (2.4)	15 (14)	34 (4.7)
2	Craniotomy Age > 17 W/O CC	367 (53.0)	61.1 (15.1)	1.7 (1.1)	5.2 (2.0)	6 (5)	9 (1.3)
576	Septicemia Age > 17	327 (48.9)	72.8 (15.3)	4.9 (1.7)	4.3 (2.2)	14 (17)	80 (12.0)
353	Pelvic Evisceration, Radical Hysterectomy & Radical Vulvectomy	584 (100.0)	59.9 (12.8)	1.8 (1.3)	5.1 (1.4)	4 (3)	9 (1.5)
203	Malignancy of Hepatobiliary System or Pancreas	237 (45.8)	70.7 (11.6)	2.8 (1.5)	3.8 (1.7)	7 (7)	3 (0.6)
260	Subtotal Mastectomy for Malignancy W/O CC	515 (100.0)	62.4 (13.1)	1.1 (0.3)	2.8 (0.8)	2 (0)	0 (0)
570	Major Small & Large Bowel Procedures W CC Without Major Gastrointestinal Diagnosis	341 (68.2)	66.6 (12.9)	3.9 (1.7)	5.8 (1.6)	8 (5)	0 (0)
14	Intracranial Hemorrhage or Cerebral Infarction	200 (41.6)	73.7 (14.3)	3.5 (1.8)	4.5 (2.1)	7 (9)	42 (8.7)
311	Transurethral Procedures W/O CC	138 (29.2)	69.1 (13.9)	1.1 (0.4)	3.1 (1.5)	3 (2)	11 (2.3)
75	Major Chest Procedures	252 (53.4)	64.9 (14.1)	1.5 (1.2)	4.0 (1.5)	4 (2)	8 (1.7)
171	Other Digestive System O.R. Procedures W/O CC	376 (82.1)	61.4 (13.9)	1.8 (0.8)	3.3 (1.3)	3 (2)	0 (0)
500	Back & Neck Procedures Except Spinal Fusion W/O CC	202 (45.9)	61.5 (15.7)	1.2 (0.7)	2.5 (1.4)	3 (2)	7 (1.6)
544	Major Joint Replacement or Reattachment of Lower Extremity	258 (60.8)	73.9 (11.2)	1.4 (1.0)	2.9 (1.7)	5.5 (4)	21 (5.0)
357	Uterine & Adnexa Procedures For Ovarian Or Adnexal Malignancy	400 (100.0)	55.6 (14.9)	2.5 (1.5)	5.7 (1.6)	4 (3)	2 (0.5)
87	Pulmonary Edema & Respiratory Failure	213 (55.2)	79.0 (13.8)	5.1 (1.6)	4.6 (2.0)	10 (10)	24 (6.2)

*Abbreviations*: DRG, diagnosis-related group; SD, standard deviation; LOS, length of stay; IQR, interquartile range; W/O, without; CC, complications; O.R., operating room; C.D.E., Common Duct Exploration; W, with

Mean age varied widely across diagnostic categories, ranging from 46.6 years among hospitalizations classified as *O.R. Procedures for Obesity* (DRG 288) to 79.0 years among hospital admissions for *Pulmonary Edema & Respiratory Failure* (DRG 87). Marked variability was also observed in sex distribution, with the proportion of female patients ranging from 29.2% in *Transurethral Procedures W/O CC* (DRG 311) to 100% in sex-specific gynecological DRGs. The burden of comorbidities and the number of documented medical procedures showed substantial heterogeneity across DRGs, reflecting differences in baseline clinical profiles and care pathways.

Median LOS demonstrated pronounced dispersion across DRGs, ranging from 2 days in short-stay surgical admissions to 15 days in medical DRGs characterized by higher clinical complexity. Overall, 263 hospitalizations (1.9%) exceeded the DRG-specific LOS threshold. The proportion of LOS outliers varied markedly across DRGs, with the highest percentages observed in *Septicemia Age > 17* (DRG 576; 12.0%), *Intracranial Hemorrhage or Cerebral Infarction* (DRG 14; 8.7%), *Pulmonary Edema & Respiratory Failure* (DRG 87; 6.2%), and *Major Joint Replacement or Reattachment of Lower Extremity* (DRG 544; 5.0%).

### NDs and NAs in the study sample and intra-DRG variability in nursing complexity (Objective *C*)

Overall, nursing complexity was characterized by a wide distribution of NDs and NAs across the sample, with substantial variability both between and within DRGs.

A total of 57,543 NDs were identified on admission in the study population, corresponding to a mean of 4.1 (SD: 2.6) NDs per patient. Overall, 34 distinct CCC diagnostic labels were documented. In the overall sample, the most frequently recorded ND was *Infection Risk* (11466; 19.9%), followed by *Fall Risk* (9727; 16.9%) and *Acute Pain* (5889; 10.2%).

A total of 293,161 NAs were documented, resulting in a median of 17 (IQR: 10) NAs per hospitalization. The most frequently recorded NA was *Assessment of patient dependency through objective examination* (12449; 4.2%), followed by *Patient identification and application of an ID wristband* (12258; 4.2%) and *Assessment and monitoring of nutritional and hydration status* (12207; 4.2%).

The distribution of NDs and NAs varied across DRGs, with each DRG exhibiting a distinct prevalence profile. Detailed DRG-specific distributions and main rankings of NDs and NAs are reported in the Supplementary Materials (Supplementary File 2).

**Fig. 2:**
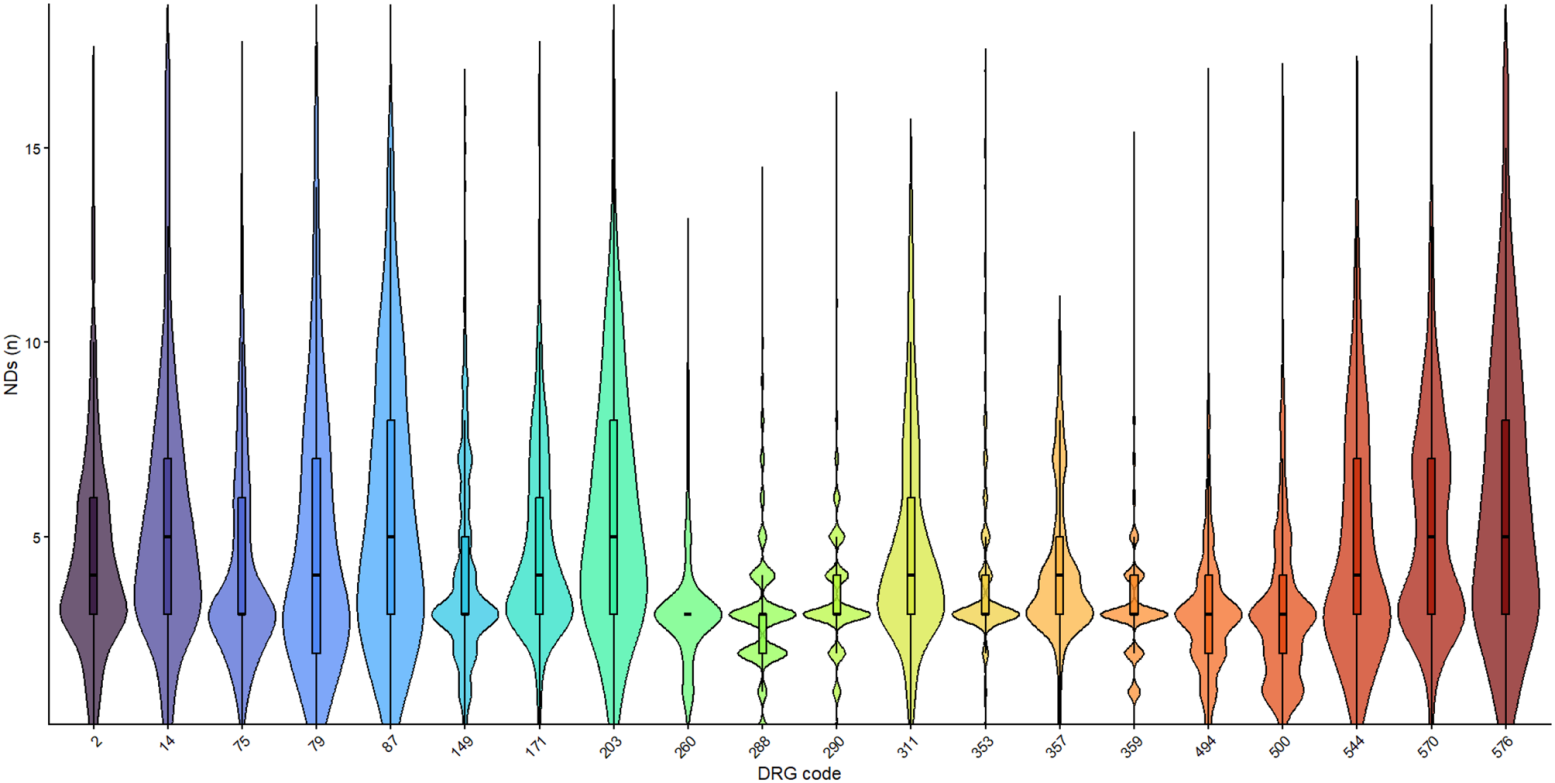
Distribution of NDs within and across DRGs. Note: Violin plots illustrating within- and between-DRG variability in NDs. For each DRG, the violin plot depicts the distribution of NDs; the embedded central marker represents the mean and the standard deviation, and individual points correspond to observed patient-level values across the entire distribution. DRG codes: DRG 2, Craniotomy Age > 17 W/O CC; DRG 14, Intracranial Hemorrhage or Cerebral Infarction; DRG 75, Major Chest Procedures; DRG 79; Respiratory Infections & Inflammations Age > 17 W CC; DRG 87, Pulmonary Edema & Respiratory Failure; DRG 149, Major Small & Large Bowel Procedures W/O CC; DRG 171, Other Digestive System O.R. Procedures W/O CC; DRG 203, Malignancy of Hepatobiliary System or Pancreas; DRG 260, Subtotal Mastectomy for Malignancy W/O CC; DRG 288, O.R. Procedures for Obesity; DRG 290, Thyroid Procedures; DRG 311, Transurethral Procedures W/O CC; DRG 353, Pelvic Evisceration, Radical Hysterectomy & Radical Vulvectomy; DRG 357, Uterine & Adnexa Procedures for Ovarian or Adnexal Malignancy; DRG 359, Uterine & Adnexa Procedures for non-Malignancy W/O CC; DRG 494, Laparoscopic Cholecystectomy W/O C.D.E. W/O CC, DRG 500, back & Neck Procedures Except Spinal Fusion W/O CC; DRG 544, Major Joint Replacement or Reattachment of Lower Extremity; DRG 570, Major Small & Large Bowel Procedures W CC without Major Gastrointestinal Diagnosis; DRG 576, Septicemia Age > 17. Abbreviations: DRG, diagnosis-related group; NDs, nursing diagnoses

**Fig. 3:**
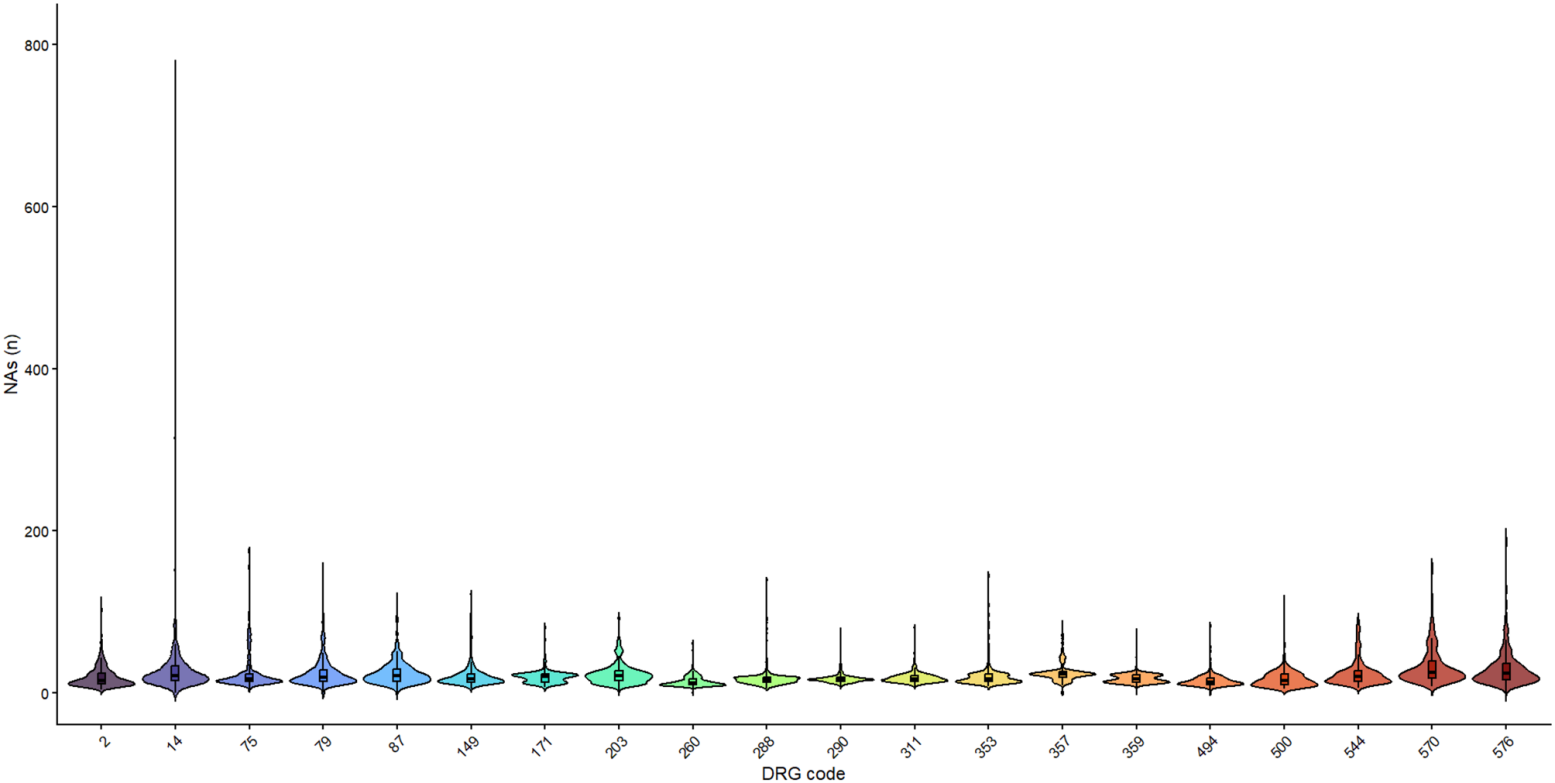
Distribution of NAs within and across DRGs. Note: Violin plots illustrating within- and between-DRG variability in NAs. For each DRG, the violin plot depicts the distribution of NAs; the embedded boxplot represents the median and the interquartile range, and individual points correspond to observed patient-level values across the entire distribution. DRG codes: DRG 2, Craniotomy Age > 17 W/O CC; DRG 14, Intracranial Hemorrhage or Cerebral Infarction; DRG 75, Major Chest Procedures; DRG 79; Respiratory Infections & Inflammations Age > 17 W CC; DRG 87, Pulmonary Edema & Respiratory Failure; DRG 149, Major Small & Large Bowel Procedures W/O CC; DRG 171, Other Digestive System O.R. Procedures W/O CC; DRG 203, Malignancy of Hepatobiliary System or Pancreas; DRG 260, Subtotal Mastectomy for Malignancy W/O CC; DRG 288, O.R. Procedures for Obesity; DRG 290, Thyroid Procedures; DRG 311, Transurethral Procedures W/O CC; DRG 353, Pelvic Evisceration, Radical Hysterectomy & Radical Vulvectomy; DRG 357, Uterine & Adnexa Procedures for Ovarian or Adnexal Malignancy; DRG 359, Uterine & Adnexa Procedures for non-Malignancy W/O CC; DRG 494, Laparoscopic Cholecystectomy W/O C.D.E. W/O CC, DRG 500, back & Neck Procedures Except Spinal Fusion W/O CC; DRG 544, Major Joint Replacement or Reattachment of Lower Extremity; DRG 570, Major Small & Large Bowel Procedures W CC without Major Gastrointestinal Diagnosis; DRG 576, Septicemia Age > 17. Abbreviations: DRG, diagnosis-related group; NAs, nursing actions

Marked variability in nursing complexity was observed not only between DRGs, but also—most prominently—within individual DRGs. Among hospitalizations classified within the same DRG, both the number of NDs at admission and the number of NAs during hospitalization showed wide and overlapping distributions. Across DRGs, within-group ranges spanned from 1 to 17 NDs and from 1 to 770 NAs, indicating substantial intra-DRG heterogeneity in nursing complexity among patients classified as medically homogeneous by administrative criteria. Individual-level dispersion of nursing complexity is illustrated in Table 3 and in the violin plots presented in Figs. 2 and 3.

**Table 3:** Nursing complexity indicators and variability by single DRG

DRG data		Nursing Data			
		NDs		NAs	
**DRG code**	**DRG description**	**Mean (SD)**	**Range** **(min-max)**	**Median (IQR)**	**Range** **(min-max)**
359	Uterine & Adnexa Procedures for Non-Malignancy W/O CC	3.2 (1.5)	1–15	17 (9)	1–76
290	Thyroid Procedures	3.4 (1.6)	1–16	16 (5)	6–78
149	Major Small & Large Bowel Procedures W/O CC	4.0 (2.4)	1–16	17 (10)	5–122
288	O.R. Procedures for Obesity	3.0 (1.8)	1–14	16 (6)	5–140
494	Laparoscopic Cholecystectomy W/O C.D.E. W/O CC	3.1 (1.7)	1–16	13 (8)	1–83
79	Respiratory Infections & Inflammations Age > 17 W CC	4.9 (3.4)	1–17	19 (14)	1–154
2	Craniotomy Age > 17 W/O CC	4.5 (2.6)	1–16	15 (13)	4–112
576	Septicemia Age > 17	6.0 (3.7)	1–17	24 (20)	1–192
353	Pelvic Evisceration, Radical Hysterectomy & Radical Vulvectomy	3.8 (2.0)	1–17	17 (9)	8–145
203	Malignancy of Hepatobiliary System or Pancreas	5.6 (3.1)	1–17	21 (12)	3–93
260	Subtotal Mastectomy for Malignancy W/O CC	3.2 (1.6)	1–12	12 (7)	1–61
570	Major Small & Large Bowel Procedures W CC Without Major Gastrointestinal Diagnosis	5.4 (2.8)	1–17	25 (21)	8–154
14	Intracranial Hemorrhage or Cerebral Infarction	5.5 (3.4)	1–17	21 (18)	1–770
311	Transurethral Procedures W/O CC	4.8 (2.7)	1–14	17 (7)	8–81
75	Major Chest Procedures	4.2 (2.6)	1–16	17 (9)	5–175
171	Other Digestive System O.R. Procedures W/O CC	4.7 (2.2)	1–16	20 (10)	7–81
500	Back & Neck Procedures Except Spinal Fusion W/O CC	3.1 (2.1)	1–16	15 (14)	5–113
544	Major Joint Replacement or Reattachment of Lower Extremity	4.7 (3.0)	1–15	20 (14)	6–91
357	Uterine & Adnexa Procedures For Ovarian Or Adnexal Malignancy	4.1 (1.9)	1–10	23 (7)	1–85
87	Pulmonary Edema & Respiratory Failure	5.6 (3.5)	1–16	21 (15)	1–115

Abbreviations: DRG, diagnosis-related group; NDs, nursing diagnoses; NAs, nursing actions; SD, standard deviation; IQR, interquartile range; W/O, without; CC, complications; O.R., operating room; C.D.E., Common Duct Exploration; W, with

### Nursing complexity according to DRG-specific LOS threshold status (Objective *D*)

Hospitalizations exceeding the DRG-specific LOS thresholds (LOS outliers; *n* = 263) showed higher nursing complexity than hospitalizations remaining within expected LOS limits (*n* = 13906).

With respect to NDs, LOS outliers had a higher mean number of NDs (mean = 7.5; SD = 4.3) compared with hospitalizations within the LOS threshold (mean = 4.0; SD = 2.5). The difference was statistically significant (Welch’s *t*(265.2) = −13.0, *p* < 0.001) and large in magnitude (Cohen’s *d* = 1.0; 95% CI 0.9–1.1), indicating a substantial difference in ND counts between the two groups.

**Fig. 4:**
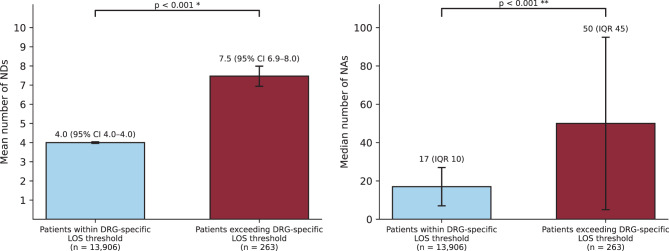
Nursing complexity according to DRG-specific LOS threshold status. Note: Mean number of NDs assessed at admission (left panel) and median number of NAs recorded during hospitalization (right panel), stratified by hospitalizations within and exceeding DRG-specific LOS thresholds. Bars represent 95% confidence intervals for group means (NDs). For NAs, boxplots depict group medians, and whiskers represent the interquartile range. Hospitalizations exceeding the LOS threshold showed higher values for both indicators (*p* < 0.001, * *t*-test; ** Mann-Whitney *U* test). Abbreviations: DRG, diagnosis-related group; LOS, length of stay; NDs, nursing diagnoses; CI, confidence interval; NAs, nursing actions; IQR, interquartile range

Similarly, the number of NAs recorded during hospitalization was higher among LOS outliers. Given the markedly skewed distribution of NAs, comparisons were conducted using a non-parametric approach (see Methods). Hospitalizations exceeding the DRG-specific LOS threshold showed a higher median number of NAs (median = 50; IQR = 45) compared with hospitalizations within expected LOS limits (median = 17; IQR = 10). The difference in distributions was statistically significant (Mann-Whitney *U* = 311249, Z = −23.1, *p* < 0.001; *r* = 0.19), with LOS outliers exhibiting higher rank values (mean rank = 12854.5 vs. 6975.9). These differences are illustrated in Fig. 4.

### Association between DRG weight and nursing complexity (Objective *E*)

Linear regression analyses showed that DRG weight was significantly associated with nursing complexity indicators but explained only a minimal proportion of their variability.

When NDs were entered as the dependent variable, the model showed a significant association (β = 0.140, *p* < 0.001), explaining 1.97% of the variance (R^2^ = 0.020; adjusted R^2^ = 0.020).

Similarly, when NAs were entered as the dependent variable, a significant association was observed (β = 0.100, *p* < 0.001), accounting for 1.0% of the variance (R^2^ = 0.010; adjusted R^2^ = 0.010). Regression coefficients and model statistics are reported in Table 4.

**Table 4:** Linear regression models examining DRG weight as a predictor of nursing complexity

Dependent Variable	B	SE	Standardized β	**R**^**2**^	**Adjusted R**^**2**^	*p***-value**
NDs counts	0.574	0.034	0.140	0.020	0.020	<0.001
NAs counts	3.182	0.221	0.100	0.010	0.010	<0.001

Note: DRG weight was entered as the independent variable in both models

Abbreviations: DRG, diagnosis-related group; B, unstandardized regression coefficient; SE, standard error; β, standardized regression coefficient; R^2^, coefficient of determination; NDs, nursing diagnoses; NAs, nursing actions

In multivariable models adjusting for age, sex, number of medical diagnoses, and number of medical procedures, DRG weight remained statistically significant for both NDs and NAs, with small standardized coefficients (NDs: β = 0.030, *p* = 0.001; NAs: β = −0.018, *p* = 0.040). Patient-level clinical variables showed higher standardized coefficients, particularly the number of medical diagnoses and procedures. Overall, the models explained a greater proportion of variability compared with univariable analyses (NDs: R^2^ = 0.130; NAs: R^2^ = 0.097), while the contribution of DRG weight remained limited (Table 5).

**Table 5:** Multivariable linear regression models examining DRG weight and covariates as predictors of nursing complexity

Dependent Variable	Predictor	B	SE	Standardized β	p-value
**NDs counts**	DRG weight	0.124	0.036	0.030	0.001
	Age	0.019	0.001	0.122	<0.001
	Sex	−0.393	0.044	−0.071	<0.001
	Number of medical diagnoses	0.293	0.014	0.192	<0.001
	Number of medical procedures	0.181	0.013	0.133	<0.001
	**Model fit (R**^**2**^** / Adjusted R**^**2**^**)**	0.130 / 0.130			
**NAs counts**	DRG weight	−0.594	0.288	−0.018	0.040
	Age	0.074	0.011	0.060	<0.001
	Sex	−1.784	0.361	−0.040	<0.001
	Number of medical diagnoses	1.583	0.115	0.130	<0.001
	Number of medical procedures	2.283	0.106	0.209	<0.001
	**Model fit (R**^**2**^** / Adjusted R**^**2**^**)**	0.097 / 0.097			

Note: Multivariable linear regression models included DRG weight, age, sex, number of medical diagnoses, and number of medical procedures as independent variables

Abbreviations: DRG, diagnosis-related group; B, unstandardized regression coefficient; SE, standard error; β, standardized regression coefficient; R^2^, coefficient of determination; NDs, nursing diagnoses; NAs, nursing actions

## Discussion

This study explored variability in nursing complexity within the most prevalent DRGs in an adult acute-care population using standardized NDs and NAs documented in routine practice. Overall, the findings show that DRG-based classification, as currently implemented, does not adequately explain the observed variability in nursing complexity and care delivered to patients, which remains largely unaccounted for within current reimbursement frameworks.

Three main findings emerge from this study. First, nursing complexity differed across the DRGs examined, indicating that medically defined case-mix categories are associated with distinct nursing care demands. This finding confirms that DRGs, while primarily constructed to reflect differences in medical diagnoses, procedures, and expected resource use, are also implicitly linked to different profiles of nursing care. However, because DRG classification logic does not explicitly incorporate nursing-sensitive measures, these differences remain embedded within medical categories rather than being directly quantified or used to inform organizational planning and reimbursement decisions [16]. As a result, variation in nursing complexity across DRGs exists in practice but remains largely unrecognized at the system level [15].

Secondly, and more importantly, considerable variability in nursing complexity was observed within the same DRGs. Patients classified as medically similar according to administrative criteria required markedly different counts of NDs at admission and NAs during the hospital stay. This intra-DRG heterogeneity exposes a structural limitation of DRG-based systems: a uniform DRG cost/weight does not imply uniform nursing complexity or nursing resource consumption within that DRG. In other words, what DRGs treat as administratively homogeneous often conceals profound differences in nursing demands at the bedside, differences that remain invisible in payment systems but are absorbed at the level of nursing services and ward-based staffing resources. This finding is consistent with prior evidence showing that DRGs primarily reflect medical resource consumption and fail to account for variability in nursing complexity [17, 18]. In our data, this misalignment was also reflected in the limited explanatory power of DRG weight, which accounted for only a minimal proportion of the variance in both ND and NA counts. Even after accounting for key clinical and demographic factors, DRG weight remained a weak predictor of nursing complexity. This suggests that the misalignment between DRG-based classification and nursing complexity is unlikely to be explained only by patient-level confounding but may reflect a structural limitation of the DRG system itself. From a bedside perspective, this finding reflects everyday clinical practice, where patients assigned to the same DRG may differ widely in care needs, dependency, clinical instability, and monitoring intensity [46]. Consequently, a single DRG code and its associated costs may mask substantial differences in nursing workload, care intensity, and the level of professional expertise required to safely deliver patient care. In this sense, DRGs may describe patients as similar, while nursing complexity reveals how different they truly are in their biopsychosocial responses to that medical condition.

A third key finding concerns LOS outliers. Hospitalizations exceeding DRG-specific LOS thresholds were consistently characterized by higher nursing complexity than those remaining within expected limits for each DRG. These prolonged stays were not merely longer in time, but qualitatively more demanding in terms of nursing care. However, this association should not be interpreted as indicating a unidirectional effect of nursing complexity on LOS, or of LOS on nursing complexity. Rather, the relationship is likely dynamic: greater care needs documented at admission may contribute to prolonged hospitalization, while longer stays may also generate additional NAs through ongoing monitoring, symptom management, discharge planning, and responses to evolving patient conditions. This bidirectional interplay suggests that LOS and nursing complexity should be interpreted as mutually related dimensions of the care process rather than as independent or sequential phenomena. In this perspective, extended LOS may reflect unresolved clinical, functional, or psychosocial complexity—dimensions that are largely addressed through sustained and adaptive nursing involvement throughout the hospitalization [25].

Taken together, these results challenge the assumption that DRG homogeneity corresponds to uniform nursing complexity. Instead, they suggest that nursing complexity represents an invisible yet systematic source of variability within DRGs, becoming particularly evident in prolonged hospitalizations. Crucially, our findings indicate that this invisibility should not be interpreted solely as a documentation or measurement gap, but rather as a structural feature of reimbursement models primarily organized around medical diagnoses, procedures, and expected resource use [2, 15, 17]. Within this architecture, nursing care is embedded within undifferentiated hospitalization costs, and variation in surveillance, dependency management, symptom monitoring, education, coordination, and discharge preparation remains operationally managed but not analytically or economically recognized.

This structural misalignment extends beyond issues of reimbursement accuracy and reflects a deeper disconnect between how hospital care is financed and how it is delivered. When variability in nursing complexity is not explicitly captured, nursing-intensive episodes are systematically absorbed within existing payment categories, potentially shifting the burden to ward-level organization, staffing, and skill mix without corresponding adjustment mechanisms. In this context, the invisibility of nursing complexity within DRG-based systems can be understood as an upstream structural condition contributing to broader organizational pressures, including workload imbalance, staffing strain, missed nursing care, intention to leave, and reduced capacity to sustain quality and safety initiatives [47–49]. In this sense, the invisibility of nursing complexity within DRG-based systems may not be a marginal technical issue, but a structural contributor to the pressures currently experienced in nursing care delivery worldwide. From a policy perspective, these findings suggest that integrating standardized nursing data into case-mix systems is not only a measurement refinement, but a necessary step toward aligning reimbursement with the structure and intensity of inpatient care delivery, including through nursing intensity weights, add-on payments, or complementary resource-allocation models [15, 23, 50, 51].

The lack of adjustment for within-DRG variability in nursing complexity raises important questions about the alignment between payment systems and care demands. From a policy standpoint, DRG-based prospective payment can generate what have been described as perverse incentives within healthcare payment systems [52]. Because reimbursement is largely fixed per episode, hospitals may be incentivized to manage operating margins by adjusting labor costs, including nursing hours per patient, through higher nurse-to-patient ratios or skill-mix dilution. When nursing care needs vary substantially within the same DRG—as observed in this study—this financing structure may systematically shift the cost of nursing-intensive episodes to wards and nursing services (i.e., cross-subsidization within DRGs), without a commensurate payment adjustment.

Separating nursing care from the standard DRG cost weight—either through nursing intensity weights, an add-on payment, or a case-mix adjustment based on routinely documented nursing data—could help realign incentives by linking reimbursement more closely to the actual nursing resources required. However, payment redesign alone is unlikely to be sufficient; it should be paired with transparent measurement standards and quality and safety safeguards to minimize unintended consequences and ensure that additional nursing resources translate into improved staffing adequacy and patient outcomes. Importantly, routinely collecting nursing-centric data does not, by itself, translate into reimbursement change under a DRG prospective payment model; as Finkler [53] argues, progress in this area requires compelling evidence that the benefits of more accurate nursing resource information outweigh the costs and practical challenges of producing, standardizing, and auditing such data—ideally through piloted and auditable nursing-intensity adjustments before broader adoption.

Within this policy context, the use of standardized nursing terminologies and electronic nursing documentation represents a concrete opportunity to address this gap. By leveraging routinely collected NDs and NAs, hospitals and policymakers can begin to quantify nursing complexity at the patient level and integrate it into case-mix analysis, performance evaluation, and resource planning [15].

### Implications for policy and nursing practice

These findings support the need for greater alignment between reimbursement mechanisms and the structure of inpatient care delivery. Policymakers and health systems funders could consider incorporating nursing-sensitive indicators into case-mix refinement strategies or complementary adjustment mechanisms, particularly for DRGs characterized by high intra-group variability. At the hospital level, nursing complexity metrics derived from standardized documentation may inform staffing models, skill-mix decisions, and targeted interventions for patients at risk of prolonged or resource-intensive hospitalizations.

### Limitations

This study has limitations that should be acknowledged. The findings should be interpreted in light of the single-center setting in Rome, Italy, which may limit transferability to other healthcare systems and organizational contexts. Although the hospital operates within the Italian National Health Service, where DRG-based reimbursement is nationally applied, it was not intended to be representative of all hospitals using DRGs in Italy, and transferability may therefore be limited to comparable hospital settings. The study also focused on the most prevalent DRGs of the study year; less frequent but highly complex DRGs warrant further investigation. Although patient and hospitalization characteristics were described by DRG, the present study did not analyze nursing complexity according to individual patient-level characteristics or model their independent contribution to NDs and NAs. Therefore, part of the observed intra-DRG variability may reflect unmeasured or insufficiently modelled patient heterogeneity, including differences in demographic, clinical, functional, cognitive, psychosocial, or dependency-related characteristics.

In addition, the requirement for study-specific informed consent, mandated by the Ethics Committee, may have introduced a potential selection bias, as non-contactable or non-consenting patients could not be included and no comparative analyses with excluded cases were feasible.

As a retrospective study based on routinely collected data, the findings may be influenced by inherent limitations of secondary data use, including potential variability in documentation practices, coding accuracy, completeness, classification validity, and reliability across professionals, wards, and clinical contexts. These limitations are particularly relevant when routinely collected administrative and clinical data are used for health services research, as such data are primarily generated for clinical, organizational, or reimbursement purposes rather than for research, and may therefore present known issues related to data quality, completeness, and coding validity [33]. Similarly, standardized nursing documentation may be affected by differences in nurses’ diagnostic reasoning, familiarity with standardized nursing terminologies, and accuracy in matching patient data with appropriate NDs. Previous studies have shown that the accuracy of NDs may vary and that diagnostic reasoning, critical thinking, professional experience, and the use of clinical decision support systems can influence the quality and consistency of nursing diagnostic documentation [54–57]. Although the PAI and its embedded clinical decision support system may have contributed to greater consistency across settings and over time, the use of standardized electronic documentation and nurse-confirmed entries cannot fully exclude measurement error, under-documentation, over-documentation, or variability in the validity and reliability of recorded NDs and NAs [38, 54].

Nurse-level and ward-level organizational variables, including staffing levels, skill mix, and nurse-to-patient ratios, were not available in the dataset; therefore, their potential influence on documentation patterns and observed variability in nursing complexity could not be examined. In addition, no data were available on nurses’ demographic characteristics, years of clinical experience, educational background, specific training in standardized nursing terminology, or diagnostic expertise. This is an important limitation because variability in nurses’ diagnostic skills may influence the number and type of NDs and NAs recorded independently of patients’ actual care needs [54], although the institutional commitment to ongoing professional development, aligned with JCI standards [32], may have contributed to greater consistency in diagnostic reasoning and documentation practices.

### Recommendations for further research

Future research should explore the integration of nursing complexity measures into predictive models of LOS, adverse outcomes, and resource utilization, as well as their potential role in informing DRG refinement or alternative payment models. Health systems funders and hospital leaders can use standardized nursing terminologies and electronic nursing documentation to quantify variability in nursing complexity within and across DRGs and incorporate it into budgeting and workforce planning.

Future studies should also incorporate nurse-level and ward-level organizational variables—such as staffing levels, skill mix, and nurse-to-patient ratios—to better understand how nursing complexity and documentation patterns relate to care delivery and resource use within DRG-based systems.

Multicenter studies and cross-national comparisons are needed to assess the transferability of our findings across different hospital settings, staffing models, reimbursement frameworks, and workforce policies.

Further work is also needed to better understand how standardized nursing documentation is generated in clinical practice, including the interplay between decision support systems and nurses’ clinical judgment, in order to more accurately interpret observed documentation patterns and strengthen the validity of nursing-sensitive indicators in health services research.

## Conclusions

Within DRG-based reimbursement systems, nursing complexity represents a critical but largely invisible component of inpatient care. This study shows that nursing complexity varies not only across DRGs but also within the same DRG and is particularly elevated in hospitalizations exceeding expected LOS thresholds.

Incorporating patient-level nursing complexity measures into case-mix classification is a necessary first step toward more accurate, equitable, and sustainable health care financing and workforce planning.

## Electronic supplementary material

Below is the link to the electronic supplementary material.

Supplementary Material 1

Supplementary Material 2

## Data Availability

The datasets generated and/or analyzed during the current study are not publicly available due to institutional data protection policies and ethical restrictions. De-identified data may be available from the corresponding author on reasonable request, subject to approval by the relevant institutional authorities and in compliance with applicable data protection regulations.

## Abbreviations

CCC: Clinical Care Classification
CI: Confidence interval
DRG: Diagnosis-related group
HDR: Hospital Discharge Register
ICD-9-CM: International Classification of Diseases, Ninth Revision, Clinical Modification
ICT: Information and Communication Technology
INNCP: Italian Nomenclature of Nursing Care Performance
IQR: Interquartile range
JCI: Joint Commission International
LOS: Length of stay
MDC: Major diagnostic category
NA: Nursing action
ND: Nursing diagnosis
PAI: Professional Assessment Instrument
RECORD: REporting of studies Conducted using Observational Routinely-collected health Data
SD: Standard deviation
