# Supplementary material for: Hidden nursing complexity within diagnosis-related groups (DRGs): a one-year retrospective study of standardized nursing diagnoses and actions among adult hospitalizations in Italy
Source: BMC Nurs. 2026 Jun 25;25:587. doi: 10.1186/s12912-026-04806-6 (PMC13332606; doi:10.1186/s12912-026-04806-6)
Supplement: Supplementary file 1 — Supplementary Material 1 [file 12912_2026_4806_MOESM1_ESM.docx]

**Supplementary File 1.** Distribution of hospitalizations by admitting unit

| **Admitting unit** | **N** | **%** | **Clinical Area** |
| --- | --- | --- | --- |
| Cardiac Surgery | 1 | 0.0 | S |
| Cardiology | 1 | 0.0 | M |
| Cardiac Electrophysiology | 1 | 0.0 | M |
| Cardiac Intensive Care | 5 | 0.0 | ICU |
| Heart Failure Unit | 1 | 0.0 | M |
| Cardiology Sub-Intensive Care | 4 | 0.0 | ICU |
| Nemo Clinical Center (Adults) | 13 | 0.1 | M |
| Nemo Clinical Center (Pediatrics) | 2 | 0.0 | M |
| Colorectal Center | 520 | 3.7 | S |
| Abdominal Surgery | 90 | 0.6 | S |
| Emergency Surgery | 176 | 1.2 | S |
| Digestive Surgery | 281 | 2.0 | S |
| Endocrine Surgery | 274 | 1.9 | S |
| Endocrine and Metabolic Surgery | 2079 | 14.7 | S |
| General Surgery | 160 | 1.1 | S |
| General Surgery 2 | 40 | 0.3 | S |
| General and Liver Transplant Surgery | 140 | 1.0 | S |
| Hepato-Biliary Surgery | 230 | 1.6 | S |
| Gynecological Surgery | 3 | 0.0 | S |
| Maxillofacial Surgery | 2 | 0.0 | S |
| Pediatric Surgery | 1 | 0.0 | S |
| Peritoneal and Retroperitoneal Surgery | 410 | 2.9 | S |
| Plastic Surgery | 26 | 0.2 | S |
| Breast Surgery | 512 | 3.6 | S |
| Thoracic Surgery | 454 | 3.2 | S |
| Vascular Surgery | 3 | 0.0 | S |
| Spine Surgery | 119 | 0.8 | S |
| Internal Medicine Unit 2 (CIC) | 83 | 0.6 | M |
| Internal Medicine Unit 3 (CIC) | 14 | 0.1 | M |
| Urology Clinic | 461 | 3.3 | S |
| Dermatology | 1 | 0.0 | M |
| Hematology | 12 | 0.1 | M |
| Hematology and Stem Cell Transplantation | 4 | 0.0 | M |
| Endocrinology and Diabetology | 47 | 0.3 | M |
| Surgical Digestive Endoscopy | 184 | 1.3 | S |
| Gastroenterology | 29 | 0.2 | M |
| Geriatrics | 148 | 1.0 | M |
| Gynecology | 909 | 6.4 | S |
| Gynecologic Oncology | 2841 | 20.1 | S |
| Clinical Immunology | 2 | 0.0 | M |
| Clinical Immunology (CIC) | 7 | 0.0 | M |
| Respiratory Failure Unit (CIC) | 56 | 0.4 | M |
| Infectious Diseases | 140 | 1.0 | M |
| Infectious Diseases (CIC) | 254 | 1.8 | M |
| Emergency Medicine | 134 | 0.9 | M |
| Liver Transplant Medicine | 38 | 0.3 | M |
| General Medicine | 94 | 0.7 | M |
| Internal Medicine 2 | 15 | 0.1 | M |
| Internal Medicine 2 (CIC) | 76 | 0.5 | M |
| Internal Medicine 3 (CIC) | 34 | 0.2 | M |
| Cardiovascular Internal Medicine | 75 | 0.5 | M |
| Internal Medicine and Gastroenterology | 267 | 1.9 | M |
| Internal Medicine and Pancreatic Diseases | 86 | 0.6 | M |
| Geriatric Internal Medicine | 98 | 0.7 | M |
| Geriatric Internal Medicine (CIC) | 102 | 0.7 | M |
| Nephrology | 20 | 0.1 | M |
| Neurosurgery | 935 | 6.6 | S |
| Pediatric Neurosurgery | 12 | 0.1 | S |
| Neurology | 122 | 0.9 | M |
| Stroke Unit | 182 | 1.3 | M |
| High-Intensity Neurorehabilitation | 15 | 0.1 | M |
| Medical Oncology | 111 | 0.8 | M |
| Hand Surgery and Orthopedics | 10 | 0.1 | S |
| Orthopedics and Traumatology | 318 | 2.2 | S |
| Obstetrics | 1 | 0.0 | M |
| Otorhinolaryngology | 16 | 0.1 | S |
| Obesity Unit | 27 | 0.2 | M |
| Obstetric Pathology | 13 | 0.1 | M |
| Pulmonology | 117 | 0.8 | M |
| Interventional Pulmonology | 11 | 0.1 | M |
| Proctology | 53 | 0.4 | S |
| Radiation Oncology | 39 | 0.3 | M |
| Rheumatology | 32 | 0.2 | M |
| Physical Medicine and Rehabilitation | 3 | 0.0 | M |
| Geriatric Rehabilitation | 2 | 0.0 | M |
| Intensive Care Unit | 65 | 0.5 | ICU |
| Intensive Care Unit (CIC) | 64 | 0.5 | ICU |
| Neurosurgical Intensive Care Unit | 66 | 0.5 | ICU |
| Postoperative Intensive Care Unit | 2 | 0.0 | ICU |
| Pediatric ICU and Trauma Center | 1 | 0.0 | ICU |
| Kidney Transplant Unit | 4 | 0.0 | S |
| Sports Traumatology and Surgery | 80 | 0.6 | S |
| Cognitive-Functional Unit | 86 | 0.6 | M |
| **Total** | **14169** | **100.0** | — |
| *Note:* Admitting units are classified into medical (M), surgical (S), or intensive care (ICU) areas based on the primary clinical function of the unit. The ICU category also included sub-intensive care settings.  *Abbreviations:* CIC, Columbus Center (satellite campus of the same hospital). | | | |
