# Supplementary material for: Hidden nursing complexity within diagnosis-related groups (DRGs): a one-year retrospective study of standardized nursing diagnoses and actions among adult hospitalizations in Italy
Source: BMC Nurs. 2026 Jun 25;25:587. doi: 10.1186/s12912-026-04806-6 (PMC13332606; doi:10.1186/s12912-026-04806-6)
Supplement: Supplementary file 2 — Supplementary Material 2 [file 12912_2026_4806_MOESM2_ESM.docx]

**Supplementary File 2.** DRG-specific distributions and main rankings of NDs and NAs

| **DRG Code** | **DRG Description** | | **Nursing Complexity Measures** | | | | | |
| --- | --- | --- | --- | --- | --- | --- | --- | --- |
| **359** | | **Uterine & Adnexa Procedures for Non-Malignancy W/O CC** | **NDs**  (N = 5,100) | **N** | **%** | **NAs (INNCP-coded)**  (N = 39,556) | **N** | **%** |
|  |  | | Fall Risk | 1674 | 41.8% | Post-operative nursing care | 2324 | 5.9 |
|  |  |  | Anxiety | 870 | 33.4% | Assessment and monitoring of nutritional and hydration status | 2220 | 5.6 |
|  |  |  | Acute Pain | 579 | 17.4% | Patient identification and application of an ID wristband | 2123 | 5.4 |
|  |  |  | Feeding Deficit | 518 | 11.6% | Pre-operative patient preparation | 2120 | 5.4 |
|  |  |  | Sleep Pattern Disturbance | 374 | 10.3% | Assessment of known allergies and previous adverse reactions | 2054 | 5.2 |
|  |  |  | Skin Integrity Impairment Risk | 280 | 7.5% | Assessment of patient dependency through objective examination | 1842 | 4.7 |
|  |  |  | Injury Risk | 150 | 5.6% | Admission reception and patient orientation | 1809 | 4.6 |
|  |  |  | Fluid Volume Deficit | 110 | 3.0% | Implementation of safety measures to reduce fall/injury risk (e.g., side rails, floor mats, handrails, non-slip surfaces) | 1554 | 3.9 |
|  |  |  | Body Nutrition Deficit | 105 | 2.2% | Bladder catheter management (e.g. bag change, patency control, skin care/control) | 1472 | 3.7 |
|  |  |  | Physical Mobility Impairment | 63 | 2.1% | Management of non-thoracic drains (e.g., patency checks, output monitoring, peristomal skin care) | 1411 | 3.6 |
|  |  |  | Others | 351 | 7.0% | Others | 20,627 | 52.1 |
| *Abbreviations:* DRG, diagnosis related group; W/O, without; CC, complications; NDs, nursing diagnoses; NAs, nursing actions; INNCP, Italian Nomenclature of Nursing Care Performance. | | | | | | | | |

| **DRG Code** | **DRG Description** | | **Nursing Complexity Measures** | | | | | |
| --- | --- | --- | --- | --- | --- | --- | --- | --- |
| **290** | | **Thyroid Procedures** | **NDs**  (N = 5,907) | **N** | **%** | **NAs (INNCP-coded)**  (N = 29,566) | **N** | **%** |
|  |  | | Infection Risk | 1511 | 25.6 | Post-operative nursing care | 1706 | 5.8 |
|  |  |  | Fall Risk | 1184 | 20.0 | Pre-operative patient preparation | 1639 | 5.5 |
|  |  |  | Acute Pain | 1070 | 18.1 | Patient identification and application of an ID wristband | 1591 | 5.4 |
|  |  |  | Skin Integrity Impairment Risk | 549 | 9.3 | Assessment of sleep/rest length and quality | 1575 | 5.3 |
|  |  |  | Sleep Pattern Disturbance | 545 | 9.2 | Environmental assessment, hazard mapping, and recommendations to ensure comfort and safety | 1350 | 4.6 |
|  |  |  | Body Nutrition Deficit | 209 | 3.5 | Assessment of patient dependency through objective examination | 1285 | 4.3 |
|  |  |  | Anxiety | 188 | 3.2 | Implementation of safety measures to reduce fall/injury risk (e.g., side rails, floor mats, handrails, non-slip surfaces) | 1230 | 4.2 |
|  |  |  | Fluid Volume Deficit | 153 | 2.6 | Fall/injury risk assessment (e.g., Conley scale) | 1226 | 4.1 |
|  |  |  | Injury Risk | 72 | 1.2 | Comprehensive pain assessment | 1101 | 3.7 |
|  |  |  | Fear | 59 | 1.0 | Assessment and monitoring of nutritional and hydration status | 1077 | 3.6 |
|  |  |  | Others | 367 | 6.2 | Others | 15,786 | 53.4 |
| *Abbreviations:* DRG, diagnosis related group; NDs, nursing diagnoses; NAs, nursing actions; INNCP, Italian Nomenclature of Nursing Care Performance. | | | | | | | | |

| **DRG Code** | **DRG Description** | | **Nursing Complexity Measures** | | | | | |
| --- | --- | --- | --- | --- | --- | --- | --- | --- |
| **149** | | **Major Small & Large Bowel Procedures W/O CC** | **NDs**  (N = 3,542) | **N** | **%** | **NAs (INNCP-coded)**  (N = 17,805) | **N** | **%** |
|  |  | | Infection Risk | 736 | 20.8 | Post-operative nursing care | 920 | 5.2 |
|  |  |  | Fall Risk | 632 | 17.8 | Patient identification and application of an ID wristband | 810 | 4.5 |
|  |  |  | Acute Pain | 362 | 10.2 | Assessment and monitoring of nutritional and hydration status | 752 | 4.2 |
|  |  |  | Fluid Volume Deficit | 223 | 6.3 | Pre-operative patient preparation | 723 | 4.1 |
|  |  |  | Sleep Pattern Disturbance | 217 | 6.1 | Assessment of patient dependency through objective examination | 664 | 3.7 |
|  |  |  | Anxiety | 184 | 5.2 | Assessment of sleep/rest length and quality | 663 | 3.7 |
|  |  |  | Body Nutrition Deficit | 153 | 4.3 | Assessment of known allergies and previous adverse reactions | 619 | 3.5 |
|  |  |  | Feeding Deficit | 147 | 4.2 | Implementation of safety measures to reduce fall/injury risk (e.g., side rails, floor mats, handrails, non-slip surfaces) | 538 | 3.0 |
|  |  |  | Skin Integrity Impairment Risk | 124 | 3.5 | Peripheral venous catheter management | 518 | 2.9 |
|  |  |  | Injury Risk | 108 | 3.0 | Intravenous infusion management | 517 | 2.9 |
|  |  |  | Others | 656 | 18.5 | Others | 11,081 | 62.2 |
| *Abbreviations:* DRG, diagnosis related group; W/O, without; CC, complications; NDs, nursing diagnoses; NAs, nursing actions; INNCP, Italian Nomenclature of Nursing Care Performance. | | | | | | | | |

| **DRG Code** | **DRG Description** | | **Nursing Complexity Measures** | | | | | |
| --- | --- | --- | --- | --- | --- | --- | --- | --- |
| **288** | | **O.R. Procedures for Obesity** | **NDs**  (N = 2,382) | **N** | **%** | **NAs (INNCP-coded)**  (N = 13,458) | **N** | **%** |
|  |  | | Infection Risk | 672 | 28.2 | Post-operative nursing care | 768 | 5.7 |
|  |  |  | Fall Risk | 525 | 22.0 | Patient identification and application of an ID wristband | 753 | 5.6 |
|  |  |  | Acute Pain | 341 | 14.3 | Pre-operative patient preparation | 733 | 5.4 |
|  |  |  | Sleep Pattern Disturbance | 193 | 8.1 | Assessment of sleep/rest length and quality | 664 | 4.9 |
|  |  |  | Skin Integrity Impairment Risk | 155 | 6.5 | Assessment and monitoring of nutritional and hydration status | 602 | 4.5 |
|  |  |  | Fluid Volume Deficit | 68 | 2.9 | Environmental assessment, hazard mapping, and recommendations to ensure comfort and safety | 582 | 4.3 |
|  |  |  | Anxiety | 68 | 2.9 | Assessment of patient dependency through objective examination | 557 | 4.1 |
|  |  |  | Body Nutrition Deficit | 66 | 2.8 | Fall/injury risk assessment (e.g., Conley scale) | 545 | 4.0 |
|  |  |  | Injury Risk | 56 | 2.4 | Implementation of safety measures to reduce fall/injury risk (e.g., side rails, floor mats, handrails, non-slip surfaces) | 463 | 3.4 |
|  |  |  | Physical Mobility Impairment | 36 | 1.5 | Collaboration with a physician in a specialized clinical area | 453 | 3.4 |
|  |  |  | Others | 202 | 8.5 | Others | 7,338 | 54.5 |
| *Abbreviations:* DRG, diagnosis related group; O.R., operating room; NDs, nursing diagnoses; NAs, nursing actions; INNCP, Italian Nomenclature of Nursing Care Performance. | | | | | | | | |

| **DRG Code** | **DRG Description** | | **Nursing Complexity Measures** | | | | | |
| --- | --- | --- | --- | --- | --- | --- | --- | --- |
| **494** | | **Laparoscopic Cholecystectomy W/O C.D.E. W/O CC** | **NDs**  (N = 2,302) | **N** | **%** | **NAs (INNCP-coded)**  (N = 10,961) | **N** | **%** |
|  |  | | Infection Risk | 578 | 25.1 | Post-operative nursing care | 616 | 5.6 |
|  |  |  | Fall Risk | 431 | 18.7 | Patient identification and application of an ID wristband | 609 | 5.6 |
|  |  |  | Acute Pain | 282 | 12.3 | Assessment and monitoring of nutritional and hydration status | 581 | 5.3 |
|  |  |  | Sleep Pattern Disturbance | 187 | 8.1 | Assessment of patient dependency through objective examination | 559 | 5.1 |
|  |  |  | Fluid Volume Deficit | 149 | 6.5 | Pre-operative patient preparation | 490 | 4.5 |
|  |  |  | Body Nutrition Deficit | 103 | 4.5 | Assessment of sleep/rest length and quality | 482 | 4.4 |
|  |  |  | Skin Integrity Impairment Risk | 96 | 4.2 | Assessment of known allergies and previous adverse reactions | 441 | 4.0 |
|  |  |  | Feeding Deficit | 94 | 4.1 | Peripheral venous catheter management | 411 | 3.7 |
|  |  |  | Injury Risk | 75 | 3.3 | Parenteral medication administration | 382 | 3.5 |
|  |  |  | Anxiety | 58 | 2.5 | Enteral medication administration | 375 | 3.4 |
|  |  |  | Others | 249 | 10.8 | Others | 6,015 | 54.9 |
| *Abbreviations:* DRG, diagnosis related group; W/O, without; C.D.E., Common Duct Exploration; CC, complications; NDs, nursing diagnoses; NAs, nursing actions; INNCP, Italian Nomenclature of Nursing Care Performance. | | | | | | | | |

| **DRG Code** | **DRG Description** | | **Nursing Complexity Measures** | | | | | |
| --- | --- | --- | --- | --- | --- | --- | --- | --- |
| **79** | | **Respiratory Infections & Inflammations Age >17 W CC** | **NDs**  (N = 3,532) | **N** | **%** | **NAs (INNCP-coded)**  (N = 17,378) | **N** | **%** |
|  |  | | Infection Risk | 410 | 11.6 | Assessment of sleep/rest length and quality | 814 | 4.7 |
|  |  |  | Fall Risk | 327 | 9.3 | Enteral medication administration | 731 | 4.2 |
|  |  |  | Sleep Pattern Disturbance | 274 | 7.8 | Vital signs measurement | 724 | 4.2 |
|  |  |  | Breathing Pattern Impairment | 274 | 7.8 | Parenteral medication administration | 705 | 4.1 |
|  |  |  | Acute Pain | 271 | 7.7 | Collaboration with a physician in a specialized clinical area | 661 | 3.8 |
|  |  |  | Skin Integrity Impairment Risk | 259 | 7.3 | Assessment and monitoring of nutritional and hydration status | 651 | 3.7 |
|  |  |  | Physical Mobility Impairment | 244 | 6.9 | Assessment of patient dependency through objective examination | 624 | 3.6 |
|  |  |  | Body Nutrition Deficit | 182 | 5.2 | Peripheral venous catheter management | 607 | 3.5 |
|  |  |  | Urinary Elimination Alteration | 165 | 4.7 | Oxygen therapy via face mask | 530 | 3.0 |
|  |  |  | Injury Risk | 127 | 3.6 | Bladder catheter management | 492 | 2.8 |
|  |  |  | Others | 999 | 28.3 | Others | 10,839 | 62.4 |
| *Abbreviations:* DRG, diagnosis related group; W, with; CC, complications; NDs, nursing diagnoses; NAs, nursing actions; INNCP, Italian Nomenclature of Nursing Care Performance. | | | | | | | | |

| **DRG Code** | **DRG Description** | | **Nursing Complexity Measures** | | | | | |
| --- | --- | --- | --- | --- | --- | --- | --- | --- |
| **2** | | **Craniotomy Age >17 W/O CC** | **NDs**  (N = 3,090) | **N** | **%** | **NAs (INNCP-coded)**  (N = 13,544) | **N** | **%** |
|  |  | | Fall Risk | 462 | 15.0 | Assessment of patient dependency through objective examination | 912 | 6.7 |
|  |  |  | Infection Risk | 448 | 14.5 | Pre-operative patient preparation | 814 | 6.0 |
|  |  |  | Injury Risk | 433 | 14.0 | Preparation of the patient for diagnostic procedures/examinations | 773 | 5.7 |
|  |  |  | Acute Pain | 336 | 10.9 | Implementation of safety measures to reduce fall/injury risk (e.g., side rails, floor mats, handrails, non-slip surfaces) | 769 | 5.7 |
|  |  |  | Physical Mobility Impairment | 220 | 7.1 | **Level of consciousness monitoring** | 694 | 5.1 |
|  |  |  | Skin Integrity Impairment Risk | 207 | 6.7 | Collaboration with a physician in a specialized clinical area | 630 | 4.7 |
|  |  |  | Sleep Pattern Disturbance | 127 | 4.1 | Patient identification and application of an ID wristband | 553 | 4.1 |
|  |  |  | Fluid Volume Deficit | 127 | 4.1 | Comprehensive pain assessment | 542 | 4.0 |
|  |  |  | Body Nutrition Deficit | 107 | 3.5 | Venous blood sampling (venipuncture) | 473 | 3.5 |
|  |  |  | Urinary Elimination Alteration | 71 | 2.3 | Electrocardiogram (ECG) acquisition/performance | 397 | 2.9 |
|  |  |  | Others | 552 | 17.9 | Others | 6,987 | 51.6 |
| *Abbreviations:* DRG, diagnosis related group; W/O, without; CC, complications; NDs, nursing diagnoses; NAs, nursing actions; INNCP, Italian Nomenclature of Nursing Care Performance. | | | | | | | | |

| **DRG Code** | **DRG Description** | | **Nursing Complexity Measures** | | | | | |
| --- | --- | --- | --- | --- | --- | --- | --- | --- |
| **576** | | **Septicemia Age >17** | **NDs**  (N = 4,020) | **N** | **%** | **NAs (INNCP-coded)**  (N = 20,247) | **N** | **%** |
|  |  | | Infection Risk | 460 | 11.4 | Assessment of patient dependency through objective examination | 968 | 4.8 |
|  |  |  | Fall Risk | 418 | 10.4 | Assessment of sleep/rest length and quality | 945 | 4.7 |
|  |  |  | Acute Pain | 295 | 7.3 | Assessment and monitoring of nutritional and hydration status | 879 | 4.3 |
|  |  |  | Sleep Pattern Disturbance | 293 | 7.3 | Collaboration with a physician in a specialized clinical area | 751 | 3.7 |
|  |  |  | Physical Mobility Impairment | 260 | 6.5 | Peripheral venous catheter management | 654 | 3.2 |
|  |  |  | Skin Integrity Impairment Risk | 256 | 6.4 | Implementation of safety measures to reduce fall/injury risk (e.g., side rails, floor mats, handrails, non-slip surfaces) | 630 | 3.1 |
|  |  |  | Body Nutrition Deficit | 249 | 6.2 | Bladder catheter management | 595 | 2.9 |
|  |  |  | Injury Risk | 171 | 4.3 | Skin monitoring | 590 | 2.9 |
|  |  |  | Fluid Volume Deficit | 168 | 4.2 | Parenteral medication administration | 578 | 2.9 |
|  |  |  | Urinary Elimination Alteration | 160 | 4.0 | Vital signs measurement | 576 | 2.8 |
|  |  |  | Others | 1290 | 32.1 | Others | 13,081 | 64.6 |
| *Abbreviations:* DRG, diagnosis related group; NDs, nursing diagnoses; NAs, nursing actions; INNCP, Italian Nomenclature of Nursing Care Performance. | | | | | | | | |

| **DRG Code** | **DRG Description** | | **Nursing Complexity Measures** | | | | | |
| --- | --- | --- | --- | --- | --- | --- | --- | --- |
| **353** | | **Pelvic Evisceration, Radical Hysterectomy & Radical Vulvectomy** | **NDs**  (N = 2,235) | **N** | **%** | **NAs (INNCP-coded)**  (N = 11,417) | **N** | **%** |
|  |  | | Infection Risk | 567 | 25.4 | Post-operative nursing care | 673 | 5.9 |
|  |  |  | Fall Risk | 525 | 23.5 | Assessment and monitoring of nutritional and hydration status | 642 | 5.6 |
|  |  |  | Feeding Deficit | 289 | 12.9 | Assessment of known allergies and previous adverse reactions | 599 | 5.2 |
|  |  |  | Anxiety | 216 | 9.7 | Patient identification and application of an ID wristband | 596 | 5.2 |
|  |  |  | Acute Pain | 137 | 6.1 | Pre-operative patient preparation | 585 | 5.1 |
|  |  |  | Skin Integrity Impairment Risk | 81 | 3.6 | Bladder catheter management | 570 | 5.0 |
|  |  |  | Sleep Pattern Disturbance | 79 | 3.5 | Admission reception and patient orientation | 526 | 4.6 |
|  |  |  | Fluid Volume Deficit | 58 | 2.6 | Assessment of patient dependency through objective examination | 520 | 4.6 |
|  |  |  | Body Nutrition Deficit | 39 | 1.7 | Management of non-thoracic drains (e.g., patency checks, output monitoring, peristomal skin care) | 517 | 4.5 |
|  |  |  | Injury Risk | 39 | 1.7 | Implementation of safety measures to reduce fall/injury risk (e.g., side rails, floor mats, handrails, non-slip surfaces) | 485 | 4.2 |
|  |  |  | Others | 205 | 9.2 | Others | 5,704 | 50.0 |
| *Abbreviations:* DRG, diagnosis related group; NDs, nursing diagnoses; NAs, nursing actions; INNCP, Italian Nomenclature of Nursing Care Performance. | | | | | | | | |

| **DRG Code** | **DRG Description** | | **Nursing Complexity Measures** | | | | | |
| --- | --- | --- | --- | --- | --- | --- | --- | --- |
| **203** | | **Malignancy of Hepatobiliary System or Pancreas** | **NDs**  (N = 2,918) | **N** | **%** | **NAs (INNCP-coded)**  (N = 11,859) | **N** | **%** |
|  |  | | Infection Risk | 392 | 13.4 | Assessment of sleep/rest length and quality | 563 | 4.7 |
|  |  |  | Fall Risk | 325 | 11.1 | Assessment of patient dependency through objective examination | 512 | 4.3 |
|  |  |  | Sleep Pattern Disturbance | 302 | 10.3 | Assessment and monitoring of nutritional and hydration status | 478 | 4.0 |
|  |  |  | Acute Pain | 301 | 10.3 | Preparation of the patient for diagnostic procedures/examinations | 467 | 3.9 |
|  |  |  | Fluid Volume Deficit | 214 | 7.3 | Peripheral venous catheter management | 445 | 3.8 |
|  |  |  | Body Nutrition Deficit | 203 | 7.0 | Patient identification and application of an ID wristband | 429 | 3.6 |
|  |  |  | Skin Integrity Impairment Risk | 166 | 5.7 | Collaboration with a physician in a specialized clinical area | 410 | 3.5 |
|  |  |  | Anxiety | 152 | 5.2 | Enteral medication administration | 402 | 3.4 |
|  |  |  | Constipation | 101 | 3.5 | Parenteral medication administration | 398 | 3.4 |
|  |  |  | Physical Mobility Impairment | 92 | 3.2 | Vital signs measurement | 370 | 3.1 |
|  |  |  | Others | 670 | 23.0 | Others | 7,385 | 62.3 |
| *Abbreviations:* DRG, diagnosis related group; NDs, nursing diagnoses; NAs, nursing actions; INNCP, Italian Nomenclature of Nursing Care Performance. | | | | | | | | |

| **DRG Code** | **DRG Description** | | **Nursing Complexity Measures** | | | | | |
| --- | --- | --- | --- | --- | --- | --- | --- | --- |
| **260** | | **Subtotal Mastectomy for Malignancy W/O CC** | **NDs**  (N = 1,622) | **N** | **%** | **NAs (INNCP-coded)**  (N = 7,197) | **N** | **%** |
|  |  | | Infection Risk | 436 | 26.9 | Post-operative nursing care | 539 | 7.5 |
|  |  |  | Fall Risk | 373 | 23.0 | Pre-operative patient preparation | 504 | 7.0 |
|  |  |  | Feeding Deficit | 263 | 16.2 | Assessment and monitoring of nutritional and hydration status | 503 | 7.0 |
|  |  |  | Acute Pain | 140 | 8.6 | Patient identification and application of an ID wristband | 440 | 6.1 |
|  |  |  | Anxiety | 85 | 5.2 | Assessment of known allergies and previous adverse reactions | 438 | 6.1 |
|  |  |  | Skin Integrity Impairment Risk | 58 | 3.6 | Assessment of patient dependency through objective examination | 410 | 5.7 |
|  |  |  | Sleep Pattern Disturbance | 55 | 3.4 | Admission reception and patient orientation | 390 | 5.4 |
|  |  |  | Fluid Volume Deficit | 54 | 3.3 | Implementation of safety measures to reduce fall/injury risk (e.g., side rails, floor mats, handrails, non-slip surfaces) | 344 | 4.8 |
|  |  |  | Body Nutrition Deficit | 30 | 1.8 | Urine output monitoring | 298 | 4.1 |
|  |  |  | Urinary Elimination Alteration | 25 | 1.5 | Peripheral venous catheter management | 233 | 3.2 |
|  |  |  | Others | 103 | 6.4 | Others | 3,098 | 43.0 |
| *Abbreviations:* DRG, diagnosis related group; W/O, without; CC, complications; NDs, nursing diagnoses; NAs, nursing actions; INNCP, Italian Nomenclature of Nursing Care Performance. | | | | | | | | |

| **DRG Code** | **DRG Description** | | **Nursing Complexity Measures** | | | | | |
| --- | --- | --- | --- | --- | --- | --- | --- | --- |
| **570** | | **Major Small & Large Bowel Procedures W CC Without Major Gastrointestinal Diagnosis** | **NDs**  (N = 2,673) | **N** | **%** | **NAs (INNCP-coded)**  (N = 15,854) | **N** | **%** |
|  |  | | Infection Risk | 470 | 17.6 | Post-operative nursing care | 656 | 4.1 |
|  |  |  | Fall Risk | 426 | 15.9 | Assessment and monitoring of nutritional and hydration status | 580 | 3.7 |
|  |  |  | Anxiety | 242 | 9.1 | Patient identification and application of an ID wristband | 575 | 3.6 |
|  |  |  | Acute Pain | 234 | 8.8 | Assessment of known allergies and previous adverse reactions | 501 | 3.2 |
|  |  |  | Fluid Volume Deficit | 173 | 6.5 | Assessment of patient dependency through objective examination | 493 | 3.1 |
|  |  |  | Breathing Pattern Impairment | 154 | 5.8 | Peripheral venous catheter management | 488 | 3.1 |
|  |  |  | Skin Integrity Alteration | 143 | 5.3 | Intravenous infusion management | 472 | 3.0 |
|  |  |  | Sleep Pattern Disturbance | 128 | 4.8 | Implementation of safety measures to reduce fall/injury risk (e.g., side rails, floor mats, handrails, non-slip surfaces) | 459 | 2.9 |
|  |  |  | Feeding Deficit | 109 | 4.1 | Pre-operative patient preparation | 440 | 2.8 |
|  |  |  | Body Nutrition Deficit | 99 | 3.7 | Comprehensive pain assessment | 439 | 2.8 |
|  |  |  | Others | 495 | 18.5 | Others | 10,751 | 67.8 |
| *Abbreviations:* DRG, diagnosis related group; W, with; CC, complications; NDs, nursing diagnoses; NAs, nursing actions; INNCP, Italian Nomenclature of Nursing Care Performance. | | | | | | | | |

| **DRG Code** | **DRG Description** | | **Nursing Complexity Measures** | | | | | |
| --- | --- | --- | --- | --- | --- | --- | --- | --- |
| **14** | | **Intracranial Hemorrhage or Cerebral Infarction** | **NDs**  (N = 2,637) | **N** | **%** | **NAs (INNCP-coded)**  (N = 19,015) | **N** | **%** |
|  |  | | Infection Risk | 298 | 11.3 | Assessment of patient dependency through objective examination | 694 | 3.6 |
|  |  |  | Fall Risk | 273 | 10.4 | Assessment of sleep/rest length and quality | 517 | 2.7 |
|  |  |  | Sleep Pattern Disturbance | 208 | 7.9 | Patient identification and application of an ID wristband | 490 | 2.6 |
|  |  |  | Physical Mobility Impairment | 206 | 7.8 | Enteral medication administration | 485 | 2.6 |
|  |  |  | Acute Pain | 200 | 7.6 | Skin monitoring | 474 | 2.5 |
|  |  |  | Injury Risk | 175 | 6.6 | Implementation of safety measures to reduce fall/injury risk (e.g., side rails, floor mats, handrails, non-slip surfaces) | 472 | 2.5 |
|  |  |  | Fluid Volume Deficit | 174 | 6.6 | Peripheral venous catheter management | 470 | 2.5 |
|  |  |  | Skin Integrity Impairment Risk | 166 | 6.3 | Assessment and monitoring of nutritional and hydration status | 462 | 2.4 |
|  |  |  | Body Nutrition Deficit | 124 | 4.7 | Fall/injury risk assessment (e.g., Conley scale) | 446 | 2.3 |
|  |  |  | Bathing/Hygiene Deficit | 94 | 3.6 | **Mobility assessment (e.g., transfers, balance, gait endurance)** | 422 | 2.2 |
|  |  |  | Others | 719 | 27.3 | Others | 14,083 | 74.1 |
| *Abbreviations:* DRG, diagnosis related group; NDs, nursing diagnoses; NAs, nursing actions; INNCP, Italian Nomenclature of Nursing Care Performance. | | | | | | | | |

| **DRG Code** | **DRG Description** | | **Nursing Complexity Measures** | | | | | |
| --- | --- | --- | --- | --- | --- | --- | --- | --- |
| **311** | | **Transurethral Procedures W/O CC** | **NDs**  (N = 2,281) | **N** | **%** | **NAs (INNCP-coded)**  (N = 8,650) | **N** | **%** |
|  |  | | Infection Risk | 419 | 18.4 | Post-operative nursing care | 473 | 5.5 |
|  |  |  | Fall Risk | 366 | 16.0 | Assessment and monitoring of nutritional and hydration status | 451 | 5.2 |
|  |  |  | Acute Pain | 253 | 11.1 | Pre-operative patient preparation | 449 | 5.2 |
|  |  |  | Urinary Elimination Alteration | 219 | 9.6 | Patient identification and application of an ID wristband | 439 | 5.1 |
|  |  |  | Constipation | 177 | 7.8 | Venous blood sampling (venipuncture) | 402 | 4.6 |
|  |  |  | Sleep Pattern Disturbance | 113 | 5.0 | **Mobility assessment (e.g., transfers, balance, gait endurance)** | 398 | 4.6 |
|  |  |  | Physical Mobility Impairment | 103 | 4.5 | Assessment of known allergies and previous adverse reactions | 386 | 4.5 |
|  |  |  | Feeding Deficit | 93 | 4.1 | Intravenous infusion management | 375 | 4.3 |
|  |  |  | Anxiety | 90 | 3.9 | Peripheral venous catheter management | 361 | 4.2 |
|  |  |  | Skin Integrity Impairment Risk | 81 | 3.6 | Bladder catheter management | 356 | 4.1 |
|  |  |  | Others | 367 | 16.1 | Others | 4,560 | 52.7 |
| *Abbreviations:* DRG, diagnosis related group; W/O, without; CC, complications; NDs, nursing diagnoses; NAs, nursing actions; INNCP, Italian Nomenclature of Nursing Care Performance. | | | | | | | | |

| **DRG Code** | **DRG Description** | | **Nursing Complexity Measures** | | | | | |
| --- | --- | --- | --- | --- | --- | --- | --- | --- |
| **75** | | **Major Chest Procedures** | **NDs**  (N = 1,969) | **N** | **%** | **NAs (INNCP-coded)**  (N = 10,997) | **N** | **%** |
|  |  | | Infection Risk | 417 | 21.2 | Enteral medication administration | 486 | 4.4 |
|  |  |  | Fall Risk | 335 | 17.0 | Parenteral medication administration | 481 | 4.4 |
|  |  |  | Acute Pain | 244 | 12.4 | Peripheral venous catheter management | 427 | 3.9 |
|  |  |  | Anxiety | 139 | 7.1 | Patient education on prescribed therapy and care plan | 395 | 3.6 |
|  |  |  | Breathing Pattern Impairment | 106 | 5.4 | Post-operative nursing care | 395 | 3.6 |
|  |  |  | Sleep Pattern Disturbance | 95 | 4.8 | Pre-operative patient preparation | 394 | 3.6 |
|  |  |  | Skin Integrity Impairment Risk | 93 | 4.7 | Patient identification and application of an ID wristband | 357 | 3.2 |
|  |  |  | Fluid Volume Deficit | 82 | 4.2 | Assessment of patient dependency through objective examination | 349 | 3.2 |
|  |  |  | Body Nutrition Deficit | 71 | 3.6 | Intravenous infusion management | 342 | 3.1 |
|  |  |  | Skin Integrity Alteration | 63 | 3.2 | Vital signs measurement | 332 | 3.0 |
|  |  |  | Others | 324 | 16.5 | Others | 7,039 | 64.0 |
| *Abbreviations:* DRG, diagnosis related group; NDs, nursing diagnoses; NAs, nursing actions; INNCP, Italian Nomenclature of Nursing Care Performance. | | | | | | | | |

| **DRG Code** | **DRG Description** | | **Nursing Complexity Measures** | | | | | |
| --- | --- | --- | --- | --- | --- | --- | --- | --- |
| **171** | | **Other Digestive System O.R. Procedures W/O CC** | **NDs**  (N = 2,132) | **N** | **%** | **NAs (INNCP-coded)**  (N = 8,754) | **N** | **%** |
|  |  | | Infection Risk | 426 | 20.0 | Post-operative nursing care | 450 | 5.1 |
|  |  |  | Fall Risk | 380 | 17.8 | Patient identification and application of an ID wristband | 434 | 5.0 |
|  |  |  | Anxiety | 261 | 12.2 | Assessment and monitoring of nutritional and hydration status | 427 | 4.9 |
|  |  |  | Acute Pain | 185 | 8.7 | Assessment of known allergies and previous adverse reactions | 416 | 4.8 |
|  |  |  | Sleep Pattern Disturbance | 127 | 6.0 | Pre-operative patient preparation | 408 | 4.7 |
|  |  |  | Feeding Deficit | 117 | 5.5 | Assessment of patient dependency through objective examination | 399 | 4.6 |
|  |  |  | Skin Integrity Impairment Risk | 103 | 4.8 | Admission reception and patient orientation | 389 | 4.4 |
|  |  |  | Fluid Volume Deficit | 100 | 4.7 | Comprehensive pain assessment | 360 | 4.1 |
|  |  |  | Breathing Pattern Impairment | 83 | 3.9 | Intravenous infusion management | 346 | 4.0 |
|  |  |  | Skin Integrity Alteration | 78 | 3.7 | Peripheral venous catheter management | 342 | 3.9 |
|  |  |  | Others | 272 | 12.8 | Others | 4,783 | 54.6 |
| *Abbreviations:* DRG, diagnosis related group; O.R., operating room; W/O, without; CC, complications; NDs, nursing diagnoses; NAs, nursing actions; INNCP, Italian Nomenclature of Nursing Care Performance. | | | | | | | | |

| **DRG Code** | **DRG Description** | | **Nursing Complexity Measures** | | | | | |
| --- | --- | --- | --- | --- | --- | --- | --- | --- |
| **500** | | **Back & Neck Procedures Except Spinal Fusion W/O CC** | **NDs**  (N = 1,375) | **N** | **%** | **NAs (INNCP-coded)**  (N = 7,415) | **N** | **%** |
|  |  | | Fall Risk | 244 | 17.7 | Pre-operative patient preparation | 474 | 6.4 |
|  |  |  | Infection Risk | 233 | 16.9 | Assessment of patient dependency through objective examination | 383 | 5.2 |
|  |  |  | Acute Pain | 165 | 12.0 | Implementation of safety measures to reduce fall/injury risk (e.g., side rails, floor mats, handrails, non-slip surfaces) | 339 | 4.6 |
|  |  |  | Injury Risk | 149 | 10.8 | Post-operative nursing care | 287 | 3.9 |
|  |  |  | Physical Mobility Impairment | 136 | 9.9 | Admission reception and patient orientation | 283 | 3.8 |
|  |  |  | Skin Integrity Impairment Risk | 71 | 5.2 | Patient identification and application of an ID wristband | 280 | 3.8 |
|  |  |  | Sleep Pattern Disturbance | 68 | 4.9 | Collaboration with a physician in a specialized clinical area | 279 | 3.8 |
|  |  |  | Fluid Volume Deficit | 44 | 3.2 | Preparation of the patient for diagnostic procedures/examinations | 255 | 3.4 |
|  |  |  | Anxiety | 38 | 2.8 | Assessment and monitoring of nutritional and hydration status | 253 | 3.4 |
|  |  |  | Bathing/Hygiene Deficit | 25 | 1.8 | Venous blood sampling (venipuncture) | 252 | 3.4 |
|  |  |  | Others | 202 | 14.7 | Others | 4,330 | 58.4 |
| *Abbreviations:* DRG, diagnosis related group; W/O, without; CC, complications; NDs, nursing diagnoses; NAs, nursing actions; INNCP, Italian Nomenclature of Nursing Care Performance. | | | | | | | | |

| **DRG Code** | **DRG Description** | | **Nursing Complexity Measures** | | | | | |
| --- | --- | --- | --- | --- | --- | --- | --- | --- |
| **544** | | **Major Joint Replacement or Reattachment of Lower Extremity** | **NDs**  (N = 2,003) | **N** | **%** | **NAs (INNCP-coded)**  (N = 10,442) | **N** | **%** |
|  |  | | Infection Risk | 275 | 13.7 | Post-operative nursing care | 474 | 4.5 |
|  |  |  | Fall Risk | 243 | 12.1 | Enteral medication administration | 409 | 3.9 |
|  |  |  | Acute Pain | 225 | 11.2 | Assessment and monitoring of nutritional and hydration status | 387 | 3.7 |
|  |  |  | Physical Mobility Impairment | 209 | 10.4 | Parenteral medication administration | 386 | 3.7 |
|  |  |  | Fluid Volume Deficit | 135 | 6.7 | Pre-operative patient preparation | 381 | 3.6 |
|  |  |  | Sleep Pattern Disturbance | 110 | 5.5 | Peripheral venous catheter management | 351 | 3.4 |
|  |  |  | Skin Integrity Impairment Risk | 95 | 4.7 | Admission reception and patient orientation | 349 | 3.3 |
|  |  |  | Anxiety | 93 | 4.6 | Assessment of patient dependency through objective examination | 337 | 3.2 |
|  |  |  | Injury Risk | 84 | 4.2 | Comprehensive pain assessment | 333 | 3.2 |
|  |  |  | Bathing/Hygiene Deficit | 70 | 3.5 | Venous blood sampling (venipuncture) | 322 | 3.1 |
|  |  |  | Others | 464 | 23.2 | Others | 6,713 | 64.3 |
| *Abbreviations:* DRG, diagnosis related group; NDs, nursing diagnoses; NAs, nursing actions; INNCP, Italian Nomenclature of Nursing Care Performance. | | | | | | | | |

| **DRG Code** | **DRG Description** | | **Nursing Complexity Measures** | | | | | |
| --- | --- | --- | --- | --- | --- | --- | --- | --- |
| **357** | | **Uterine & Adnexa Procedures For Ovarian Or Adnexal Malignancy** | **NDs**  (N = 1,642) | **N** | **%** | **NAs (INNCP-coded)**  (N = 9,733) | **N** | **%** |
|  |  | | Infection Risk | 383 | 23.3 | Post-operative nursing care | 443 | 4.6 |
|  |  |  | Fall Risk | 356 | 21.7 | Comprehensive pain assessment | 433 | 4.4 |
|  |  |  | Anxiety | 271 | 16.5 | Assessment and monitoring of nutritional and hydration status | 424 | 4.4 |
|  |  |  | Acute Pain | 109 | 6.6 | Admission reception and patient orientation | 418 | 4.3 |
|  |  |  | Sleep Pattern Disturbance | 92 | 5.6 | Bladder catheter management | 409 | 4.2 |
|  |  |  | Skin Integrity Impairment Risk | 79 | 4.8 | Patient identification and application of an ID wristband | 406 | 4.2 |
|  |  |  | Fluid Volume Deficit | 62 | 3.8 | Assessment of known allergies and previous adverse reactions | 404 | 4.2 |
|  |  |  | Skin Integrity Alteration | 61 | 3.7 | Management of non-thoracic drains (e.g., patency checks, output monitoring, peristomal skin care) | 398 | 4.1 |
|  |  |  | Breathing Pattern Impairment | 57 | 3.5 | Intravenous infusion management | 397 | 4.1 |
|  |  |  | Feeding Deficit | 53 | 3.2 | Peripheral venous catheter management | 396 | 4.1 |
|  |  |  | Others | 119 | 7.2 | Others | 5,605 | 57.6 |
| *Abbreviations:* DRG, diagnosis related group; NDs, nursing diagnoses; NAs, nursing actions; INNCP, Italian Nomenclature of Nursing Care Performance. | | | | | | | | |

| **DRG Code** | **DRG Description** | | **Nursing Complexity Measures** | | | | | |
| --- | --- | --- | --- | --- | --- | --- | --- | --- |
| **87** | | **Pulmonary Edema & Respiratory Failure** | **NDs**  (N = 2,174) | **N** | **%** | **NAs (INNCP-coded)**  (N = 9,313) | **N** | **%** |
|  |  | | Infection Risk | 239 | 11.0 | Assessment of patient dependency through objective examination | 405 | 4.3 |
|  |  |  | Fall Risk | 228 | 10.5 | Assessment of sleep/rest length and quality | 362 | 3.9 |
|  |  |  | Acute Pain | 160 | 7.4 | Assessment and monitoring of nutritional and hydration status | 348 | 3.7 |
|  |  |  | Physical Mobility Impairment | 158 | 7.3 | Vital signs measurement | 339 | 3.6 |
|  |  |  | Breathing Pattern Impairment | 156 | 7.2 | Peripheral venous catheter management | 324 | 3.5 |
|  |  |  | Skin Integrity Impairment Risk | 141 | 6.5 | Collaboration with a physician in a specialized clinical area | 313 | 3.4 |
|  |  |  | Sleep Pattern Disturbance | 124 | 5.7 | Parenteral medication administration | 302 | 3.2 |
|  |  |  | Body Nutrition Deficit | 108 | 5.0 | Enteral medication administration | 300 | 3.2 |
|  |  |  | Urinary Elimination Alteration | 108 | 5.0 | Bladder catheter management | 285 | 3.1 |
|  |  |  | Injury Risk | 99 | 4.6 | Skin monitoring | 264 | 2.8 |
|  |  |  | Others | 653 | 30.0 | Others | 6,071 | 65.2 |
| *Abbreviations:* DRG, diagnosis related group; NDs, nursing diagnoses; NAs, nursing actions; INNCP, Italian Nomenclature of Nursing Care Performance. | | | | | | | | |
